# Advances in Semiconductor Optical Amplifier Technologies for All-Optical Logic Gate Implementations: A Comprehensive Review

**DOI:** 10.3390/nano16030202

**Published:** 2026-02-04

**Authors:** Jiali Cui, Kyriakos E. Zoiros, Amer Kotb

**Affiliations:** 1 School of Chips, XJTLU Entrepreneur College (Taicang), Xi’an Jiaotong-Liverpool University, Taicang 215400, China; jiali.cui25@student.xjtlu.edu.cn; 2 Department of Electrical Engineering and Electronics, University of Liverpool, Liverpool L69 3GJ, UK; 3 Lightwave Communications Research Group, Department of Electrical and Computer Engineering, School of Engineering, Democritus University of Thrace, 671 00 Xanthi, Greece; kzoiros@ee.duth.gr; 4 Department of Physics, Faculty of Science, Fayoum University, Fayoum 63514, Egypt

**Keywords:** semiconductor optical amplifiers (SOAs), quantum-dot SOA (QD-SOA), photonic-crystal SOA (PhC-SOA), reflective SOA (RSOA), carrier-reservoir SOA (CR-SOA), nonlinear optical effects, all-optical logic gates

## Abstract

Semiconductor optical amplifiers (SOAs) are central to the development of ultrafast, low-power all-optical signal processing systems. Their strong nonlinear response, compact size, and compatibility with photonic integration platforms make them key enablers for implementing all-optical logic functions beyond the limitations of electronic switching. This review offers a comprehensive analysis of the principal SOA technologies used in all-optical logic gate implementations, including conventional bulk and quantum well SOAs, quantum dot SOAs (QD-SOAs), photonic crystal SOAs (PhC-SOAs), reflective SOAs (RSOAs), and carrier reservoir SOAs (CR-SOAs). For each architecture, we examine the carrier dynamics, gain recovery mechanisms, saturation behavior, and fabrication considerations, together with their associated nonlinear effects such as cross-gain modulation, cross-phase modulation, and four-wave mixing. We further evaluate reported implementations of key logic operations—AND, NAND, OR, NOR, XOR, and XNOR—highlighting performance trade-offs in terms of speed, extinction ratio, operational power, integration complexity, and scalability. The review concludes with current challenges and emerging research directions aimed at realizing fully integrated, high-speed, and energy-efficient all-optical logic systems based on next-generation SOA technologies.

## 1. Introduction

With the rapid advancement of next-generation information technologies, such as 5G communications, cloud computing, and quantum communications, global data traffic is experiencing exponential growth. According to Cisco’s projections, global data center traffic will reach 20.6 ZB per year by 2025, with peak rate demands exceeding the Tb/s scale [1]. Traditional electronic signal processing technologies face severe rate bottlenecks due to physical limitations imposed by electron migration velocity and RC delay. Their processing rates struggle to exceed 100 Gb/s, while also suffering from excessive power consumption and challenging thermal management constraints [2]. However, all-optical signal processing technology has emerged as the core technical solution to overcome these bottlenecks by directly performing signal modulation, logical operations, and transmission within the optical domain. This approach eliminates the additional losses and delays caused by the conversion between optical and electrical. As the fundamental building blocks of all-optical computing and all-optical signal processing, all-optical logic gates play a crucial role in performing logical operations on optical signals, signal regeneration, header recognition, routing selection, and data encryption [3]. Their performance directly determines the transmission speed, capacity, reliability, and security of all-optical networks. However, during logical operations, optical signals inevitably experience attenuation due to factors such as insertion loss and scattering. When the output signal strength of a logic gate falls below the reception sensitivity of subsequent units or the system’s noise tolerance due to attenuation, it cannot reliably drive the next-stage device and cannot ensure the determinism of Boolean logic operations, thereby causing bit errors. Therefore, amplifying the output signal to compensate for losses is a necessary condition for maintaining the correct functionality of large-scale photonic digital circuits. Against this background, the semiconductor optical amplifier (SOA) has emerged as an ideal core component for optical signal processing due to its advantages of miniaturization, low power consumption, and ease of integration [4,5]. SOA provides gain through its active waveguide medium, typically measuring around 1 mm in length, far shorter than meter-scale fiber amplifiers, making it more suitable for on-chip integration [6]. More importantly, SOA exhibits a rich array of nonlinear optical effects, such as cross-gain modulation (XGM), cross-phase modulation (XPM), and four-wave mixing (FWM) [7,8,9,10], which provides a natural physical foundation for implementing various logic operations in both the time and frequency domains.

**Figure 1:**
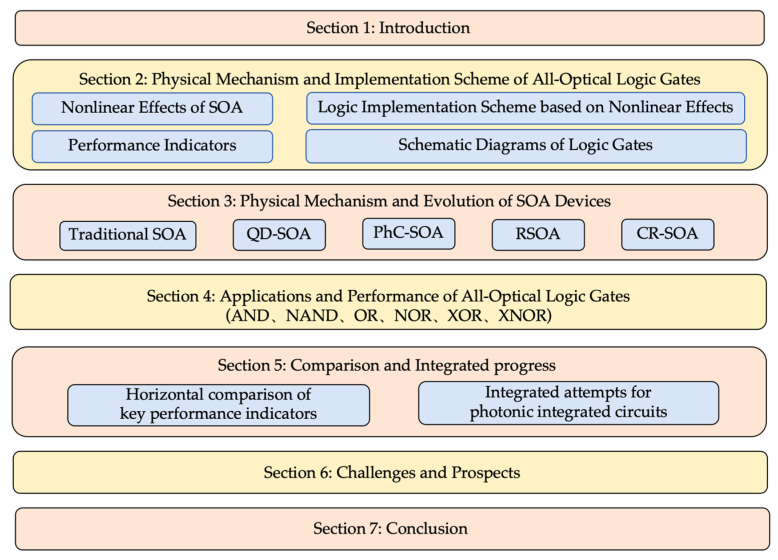
Outline of the proposed review paper.

Research on all-optical logic gates based on SOAs began in the 1990s, with its technological development divided into three key phases, tracing an evolution from proof of concept to high-performance integration. The first phase spanned from 1990 to the early 2000s, during which performance verification primarily leveraged the nonlinear effects of conventional SOAs, achieving logic operations not exceeding 40 Gb/s. However, constrained by the nanosecond-level gain recovery time of SOAs, components at this stage generally suffer from low processing speeds, bulky sizes, and low integration levels, making it difficult to meet the demands of high-speed optical communications. The second phase, spanning from the mid-2000s to the present, has seen two primary approaches emerge to enhance speed. The first involves the introduction of a revolutionary quantum dot semiconductor optical amplifier (QD-SOA), whose three-dimensional quantum confinement effect delivers outstanding characteristics such as ultrafast carrier recovery, temperature insensitivity, and low noise. This technology is pivotal for achieving high-speed all-optical logic processing at 100 Gb/s and beyond [11,12,13]. On the other hand, the photonic crystal semiconductor optical amplifier (PhC-SOA) has emerged. By leveraging the slow-light effect within photonic crystals, it significantly enhances the light with matter interactions, enabling nonlinear functionality with minimal power consumption and ultra-compact dimensions. This represents a crucial technological pathway toward ultra-high efficiency, highly integrated photonic chips, laying the foundation for the development of all-optical integrated circuits [14,15,16]. The third phase, from 2010 to the present, due to the characteristics of the reflective semiconductor optical amplifier (RSOA), such as unidirectional optical entry/exit and compact structure, has been integrated with other passive components (such as waveguides and couplers) on photonic integrated circuits. It serves as a core active component for constructing compact interferometer logic gates, enabling the realization of chip-scale all-optical processors [17]. Simultaneously, to explore integrated applications, a novel physical mechanism known as the carrier reservoir semiconductor optical amplifier (CR-SOA), which structurally optimizes a conventional SOA to overcome its gain recovery time bottleneck. By introducing an additional carrier reservoir (CR) region adjacent to the active region (AR), it rapidly replenishes depleted carriers, thereby significantly enhancing the gain recovery speed of SOA and improving the processing rate and quality factor (QF) of optical logic gates [18,19,20]. However, existing research still faces several challenges, including the management of ultrafast carrier dynamics, crosstalk control during the integration of multiple logic functions, parameter dispersion caused by process sensitivity, and core bottlenecks such as high-power consumption and system-level compatibility [21,22]. These constraints hinder the evolution from high-performance devices to stable, reliable, and mass-scalable photonic information processing systems. Therefore, this paper systematically reviews five device categories (SOAs, QD-SOAs, PhC-SOAs, RSOAs, and CR-SOAs) to elucidate their physical mechanisms and logic gate implementation schemes. By comparing their performance differences, this review aims to provide theoretical foundations for overcoming existing bottlenecks and directing optimization of device design and integration strategies, thereby advancing all-optical logic gates toward high-speed, low-power, and highly integrated practical applications. This review aims to systematically summarize the latest advancements in SOAs and their derivative device structures within the field of all-optical logic gates (AND, NAND, OR, NOR, XOR, and XNOR), with the framework structure illustrated in Figure 1.

The remainder of this paper is structured as follows: Section 2 elucidates the core physical mechanisms and implementation schemes of all-optical logic gates, including the fundamental nonlinear effects of SOAs, logic implementation schemes based on these effects, performance indicators for all-optical logic gates, and schematic diagrams of the logic gates. Section 3 introduces the physical mechanisms and evolution of SOA devices (SOAs, QD-SOAs, PhC-SOAs, RSOAs, and CR-SOAs), covering their structural characteristics, carrier dynamics, and performance advantages. Section 4 explores the specific applications and performance of these five devices in all-optical logic gates, including operation principles and optical-logic gate technological development based on SOA devices. Section 5 provides a multidimensional comparative analysis of the five devices and explores integration attempts for photonic integrated circuits. Section 6 analyzes challenges arising from existing research and outlines future development directions. Section 7 concludes with a detailed references list.

## 2. Physical Mechanism and Implementation Scheme of All-Optical Logic Gates

### 2.1. Nonlinear Effects of SOAs

The SOA is the core device for realizing all-optical logic functions. Its active region exhibits multiple nonlinear optical effects under intense optical signal injection, all mediated by dynamic carrier concentration changes during amplification. XGM, XPM, and FWM are three key nonlinear effects that provide the physical foundation for implementing all-optical logic gates.

#### 2.1.1. Cross-Gain Modulation

The cross-gain modulation (XGM) effect is based on the gain saturation characteristics of SOA. Its core mechanism lies in the fact that when a high-intensity pump beam $\lambda_{1}$ is injected into the SOA, it consumes many carriers in the active region through stimulated emission, causing an instantaneous decrease in carrier concentration. This change in carrier concentration directly modulates the gain coefficient of the semiconductor material, shifting the quasi-Fermi level and reducing the population inversion. Consequently, the macroscopic gain coefficient of the SOA decreases, entering a state of gain saturation. If, at this time, a lower power detection beam ($\lambda_{2}$) is transmitted alongside the pump beam ($\lambda_{1}$) within the SOA, the effective gain experienced by the detection beam will be directly modulated by the pump beam intensity. Consequently, the output detection light signal exhibits a logic state opposite to that of the input pump beam signal. Specifically, when the pump beam is at the high level “1”, the SOA gain is strongly suppressed, and the detection beam output power is significantly reduced. When the pump beam is at the low level “0”, the SOA gain recovers, and the detection beam output power increases. This process is directly constrained by the carrier recovery time of the SOA. Furthermore, the gain saturation induced by the pump beam significantly amplifies spontaneous emission (ASE) noise, distorting the detection beam pulse waveform [23]. Therefore, despite its simple structure, the signal quality is relatively poor. It is suitable for NOT gate or wavelength conversion applications with lower requirements for extinction ratio (ER) and noise tolerance [24].

#### 2.1.2. Cross-Phase Modulation

The cross-phase modulation (XPM) is a type of intensity-phase nonlinear transfer process in SOA, whose physical mechanism stems from the change in carrier concentration, while the physical quantity it acts upon is the refractive index of the material. According to the linewidth enhancement effect in semiconductor physics (characterized by the α factor), any change in carrier concentration will not only alter the gain but also cause a significant change in the refractive index of the material. The relationship between the two can be expressed as follows [5,25]:

(1) $$\Delta n=-\frac{\alpha }{4\pi }\lambda_{0}\Delta g,$$

where $\lambda_{0}$ represents the vacuum wavelength, and Δg is the gain variation. When a strong pump beam ($\lambda_{1}$) is injected into the SOA and changes the carrier concentration, the refractive index experienced by the co-propagating probe beam ($\lambda_{2}$) also changes accordingly, thereby accumulating a nonlinear phase shift Δ∅, whose magnitude is proportional to the propagation length and the variation in the carrier concentration [26]:

(2) $$\;\Delta \emptyset =\frac{2\pi }{\lambda }\Gamma \Delta nL,$$

Here, L represents the actuation length and Γ is the limiting factor. Different from XGM, XPM does not directly alter the intensity of the detected light. The individual phase modulation is difficult to directly detect with the photodetector, so it is usually necessary to use an interferometer structure, such as the Mach–Zehnder interferometer (MZI) interferometer or the Sagnac interferometer. By precisely controlling the phase difference between the two arms of the interferometer, the phase change can be converted into intensity change, thereby obtaining a high extinction ratio, low chirp characteristics, and a high-quality output signal without logical inversion. Therefore, XPM is the cornerstone for achieving high-performance, high-speed all-optical logic gates, but its performance is highly dependent on the stability of the interferometer structure and the precise control of the phase balance between the two arms [27].

#### 2.1.3. Four-Wave Mixing

Four-wave mixing (FWM) is a parametric mixing process in SOAs that originates from third-order nonlinear polarizability. When a strong pump light $\omega_{p}$ and a weak signal light $\omega_{s}$ are simultaneously injected into the SOA, they interfere in the time domain to form an optical beat frequency signal oscillating at the difference frequency $\Omega =\left|w_{p}-w_{s}\right|.\;$This periodically varying intensity drives the carrier concentration (N)within the active region to oscillate periodically at the same frequency Ω through the nonlinear gain mechanism. The oscillating carrier concentration consequently modulates the refractive index and gain of the material. This effect is equivalent $v_{g}$ to forming a dynamic moving grating within the SOA, propagating at a velocity with a period determined by Ω. When a strong pump light $w_{p}$ illuminates this dynamic grating formed by both pump and signal light, Bragg scattering occurs. This process generates two new frequency components, one idler $\omega_{i}=\omega_{p}+\Omega$, and a weaker conjugate signal $\omega_{c}=\omega_{s}-\Omega$ [28]. The newly generated idler utilizes the amplitude and phase information of both the pump and signal lights, enabling complex all-optical signal processing functions including wavelength conversion, phase conjugation, and signal regeneration. A significant advantage of FWM technology lies in its inherent bit rate and modulation format transparency, enabling it to process ultra-high-speed optical signals with unknown modulation formats. However, its conversion efficiency is typically lower than that of schemes based on XGM or XPM, and it is more sensitive to the polarization state between the pump and signal light, as well as phase-matching conditions. Nevertheless, FWM demonstrates unique potential for implementing all-optical logic operations, particularly for phase-encoded signal processing [29].

### 2.2. Logic Implementation Scheme Based on Nonlinear Effects

Based on these nonlinear effects, three main schemes for implementing all-optical logic gates have been derived: an XGM/XPM scheme based on a single SOA, an XPM scheme based on an interferometer, and a scheme based on an ultrafast nonlinear interferometer (UNI), which significantly enhances the overall performance of implementing logic gates [30].

#### 2.2.1. XGM/XPM Scheme Based on a Single SOA

The XGM/XPM scheme based on a single SOA employs the simplest architecture. Typically, the pump light carrying logical information and a continuous wave (CW) beam are coupled together into a single SOA via a coupler. At the output, an optical filter is used to separate the signals [31]. This approach primarily leverages XGM effects. When the pump light is a logical “1”, its carrier consumption saturates the SOA gain, thereby reducing the output intensity of the detection light to implement logic functions such as NOT gates [32]. By adjusting the input configuration, a logic OR gate can also be realized [33]. If we utilize the XPM, the SOA must be embedded within structures like Sagnac ring mirrors to form nonlinear optical ring mirrors [34]. The optical round-trip travel and interference are utilized to achieve more flexible logical functions. The core advantage of this approach lies in its extremely compact structure and low cost [35]. However, its performance is fundamentally constrained by the carrier recovery time of the SOA, limiting processing speed. XGM-based implementations introduce significant signal degradation, including output inversion, ASE noise, and pulse waveform distortion, resulting in poor signal quality. This makes it unsuitable for cascading multiple logic gates and is typically applied to principal verification or simple wavelength conversion, where speed and ER requirements are low.

#### 2.2.2. XPM Scheme Based on Interferometer

The XPM scheme based on interferometers constitutes the typical system for current high-performance all-optical logic gates. This scheme is based on MZI or delay interferometers (DI) as the basic framework and integrates SOA as the nonlinear phase modulation element in one or two of its interference arms. Its working mode is to inject the logic input signal into the SOA and modulate the phase of the continuous probe light propagating in that arm through the XPM effect. By precisely controlling the static phase difference (bias point) between the two arms of the interferometer, the two components of the CW probe undergo constructive or destructive interference at the output coupler, thereby efficiently converting the phase information into intensity output, and achieving various Boolean logic functions such as AND, OR, NOT, and XOR gates [4,36,37,38]. The core advantage of this scheme lies in being able to obtain high-ER, low-chirp, and high-quality output signals, and by adopting a differential-input design, it can effectively suppress common-mode noise. Therefore, it provides the main approach for achieving high-speed and high-fidelity all-optical logic processing. However, the performance of this scheme is extremely dependent on the consistency of the characteristics of the two SOAs, the polarization stability of the entire interferometer structure, and the long-term thermal stability, which pose severe challenges to device manufacturing and system control [39,40].

#### 2.2.3. Scheme Based on Ultrafast Nonlinear Interferometer

The scheme, based on an UNI, is an innovative interferometer structure designed to overcome the speed limitations of SOA. The core of its architecture employs a polarization controller and a differential delay line to split the input signal light into two orthogonal polarization components. These components are then passed through the same SOA sequentially after being temporally offset by a minute delay. The signal light itself acts as both pump beam and CW probe. The operational principle involves differentially comparing the distinct XPM phase shifts generated by the SOA for these temporally separated polarization components, followed by their synthesis at the output polarization interferometer to achieve logical functionality. The revolutionary aspect of this scheme is that the effective interference occurs between the nonlinear responses of the same signal pulse at different time periods of the same SOA, rather than between the responses of two independent SOAs or spatially separated arms, thus making it insensitive to the carrier recovery time of the SOA. Consequently, even using conventional bulk SOA with longer recovery times, ultra-high-speed logic operations far exceeding their gain recovery time limitations have been successfully demonstrated [41,42]. This characteristic gives the UNI scheme significant advantages when pursuing rates of 100 Gb/s and beyond. However, the implementation cost of this scheme requires extremely precise control of polarization states and accurate differential delay, and any deviation will lead to a sharp decline in interference contrast and make system debugging and maintenance more difficult.

### 2.3. Performance Evaluations Indicators

To objectively and quantitatively evaluate the performance of the signal processing unit of the all-optical logic gate based on SOA, a series of standardized evaluation indicators was adopted, including QF, ER, and signal jitter, to comprehensively cover key aspects such as signal quality, noise tolerance, and dynamic stability.

#### 2.3.1. Quality Factor

The quality factor (QF) is the core comprehensive indicator for evaluating the dynamic signal integrity and the system error code performance. This parameter is obtained by analyzing the eye diagram of the received signal, and its definition is the ratio of the difference between the average values of the logic “1” levels and the logic “0” levels to the sum of their respective noise standard deviations [43]:

(3) $$Q=\frac{\left|\mu_{1}-\mu_{0}\right|}{\sigma_{1}+\sigma_{0}},$$

Here, $\mu_{1}$ and $\mu_{0}$ represent the average signal levels of logic “1” and “0”, respectively, while $\sigma_{1}$ and $\sigma_{0}$ are the corresponding noise standard deviations. Their main sources include ASE noise, thermal noise, and fluctuations caused by the code pattern effect. The QF is directly related to the bit error rate (BER), with $BER=0.5\;erfc\left(Q/\sqrt{2}\right)$. A higher QF indicates a higher recognition degree of the signal in the presence of noise and a lower system BER. Generally, Q>6 (corresponding to $BER<10^{-9}$) is considered an acceptable threshold for high-speed optical communication systems [44]. This indicator comprehensively reflects the effects of amplitude noise, timing jitter, and inter-symbol interference.

#### 2.3.2. Noise Sensitivity and Architecture-Dependent Impact on QF

While the QF defined in Section 2.3.1 provides a unified metric for evaluating signal integrity, the relative contribution of different noise sources strongly depends on the underlying SOA architecture. In conventional bulk and quantum well SOAs, ASE noise and carrier depletion-induced pattern effects are the dominant contributors to performance degradation, particularly under high-speed operation where incomplete carrier recovery leads to pronounced inter-symbol interference [5].

Quantum dot SOAs (QD-SOAs) exhibit reduced ASE noise and improved signal robustness owing to their discrete energy states, high differential gain, and ultrafast carrier replenishment from excited states. These characteristics effectively suppress pattern-dependent noise and enable stable operation at bit rates exceeding 100 Gb/s [11,12,13,44].

In photonic crystal SOAs (PhC-SOAs), slow-light-enhanced nonlinear interactions significantly reduce the required switching power. However, increased sensitivity to fabrication-induced scattering and slow-light-related excess noise can negatively impact signal quality if dispersion and propagation loss are not carefully controlled [15,16].

Reflective SOAs (RSOAs), due to their double-pass amplification configuration, provide enhanced nonlinear efficiency but also suffer from stronger ASE accumulation and spatial carrier non-uniformity, which may degrade performance in cascaded logic configurations [5,17].

Carrier reservoir SOAs (CR-SOAs) mitigate pattern-dependent noise by rapidly replenishing carriers in the active region, thereby maintaining more stable signal quality under high-speed and long-sequence operation [18,19,20].

This architecture-dependent noise behavior explains the observed variations in QF values and underscores the importance of jointly considering carrier dynamics, ASE noise, and device structure when evaluating the performance of all-optical logic gates. 

#### 2.3.3. Extinction Ratio

The extinction ratio (ER) is a key parameter for measuring the discrimination degree of logical levels in the output optical signal. It is defined as the ratio of the minimum optical power of logic “1” to the maximum optical power of logic “0”. Its formula is as follows [45]:

(4) $$ER\;\left(dB\right)=10\;{log}_{10}\left(\frac{P_{min}^{1}}{P_{max}^{0}}\right),$$

Here, $P_{min}^{1}\;$ represents the minimum peak power of the logical “1” state under long sequence testing, while $P_{max}^{0}$ represents the maximum peak power of the logical “0” state. The higher the ER value, the greater the distinction between the logical “1” and “0”, and the better the signal quality [46]. A high ER (typically requiring more than 10 dB) is the basis for ensuring that the receiver can clearly distinguish the logical levels and directly relates to the system’s receiving sensitivity. In all-optical logic gates, polarization-dependent loss, SOA gain non-uniformity, and detector responsivity differences all affect their measured values.

#### 2.3.4. Signal Jitter

The signal jitter is a key parameter that characterizes the timing and amplitude stability of digital signals, specifically categorized into amplitude jitter and timing jitter. Amplitude jitter refers to the random power fluctuation of the logic level, and its standard deviation, $\sigma_{a},$ directly participates in the calculation of the QF. It mainly originates from the ASE noise of SOA, the gain jitter caused by the fluctuation of carrier density, and the drive power noise [47]. Timing jitter refers to the deviation of the signal transition edge from the ideal clock position. In all-optical logic gates, this is mainly caused by the nonlinear dynamics of the SOA (such as carrier heating (CH) and spectral hole burning (SHB)), as well as pulse phase distortion due to subsequent dispersion. Time jitter compresses the horizontal spread of the eye diagram and is a key factor limiting the performance of ultra-high-speed systems. Both can be quantitatively analyzed by the distribution of ‘1’ and ‘0’ levels in the statistical eye diagram or by utilizing the spectral density of phase noise [48].

### 2.4. Schematic Diagrams of Logic Gates

**Figure 2:**
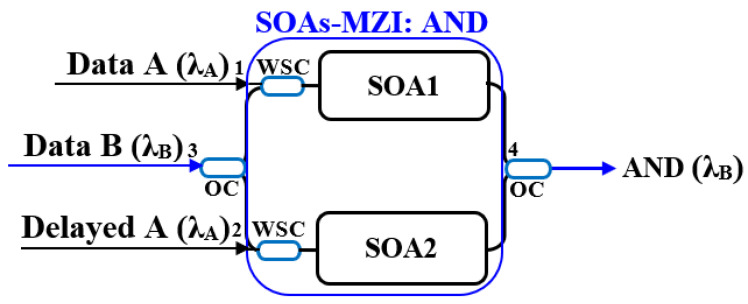
AND operation with the SOAs-MZI schematic diagram. OC: 3 dB optical coupler. WSC: wavelength selective coupler [20], “reprinted with permission from Ref. [20], licensed under CC-BY 4.0”.

**Figure 3:**
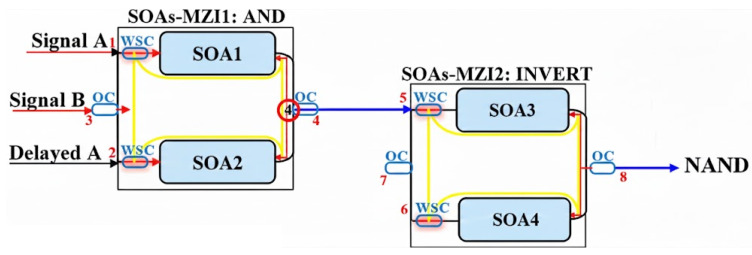
NAND operation with the SOAs-MZI schematic diagram [5], “adapted from Ref. [5], World Scientific (2015)”.

**Figure 4:**
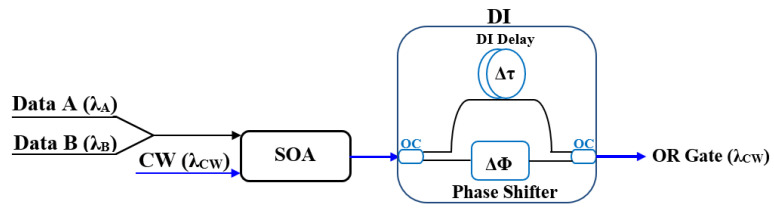
OR operation with the SOA-DI schematic diagram. CW: continuous wave [37], “reprinted with permission from Ref. [37], copyright (2016), SPIE”.

**Figure 5:**
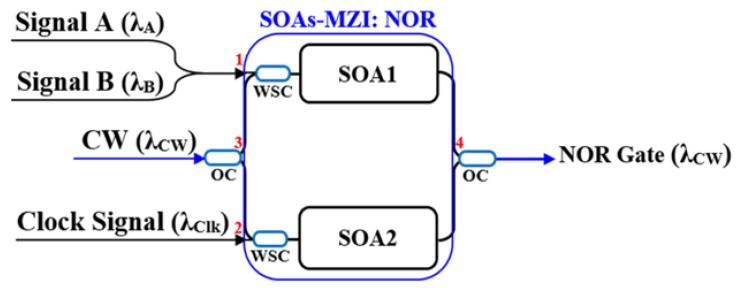
NOR operation with the SOAs-MZI schematic diagram [38], “reprinted with permission from Ref. [38], copyright (2021), Elsevier”.

**Figure 6:**
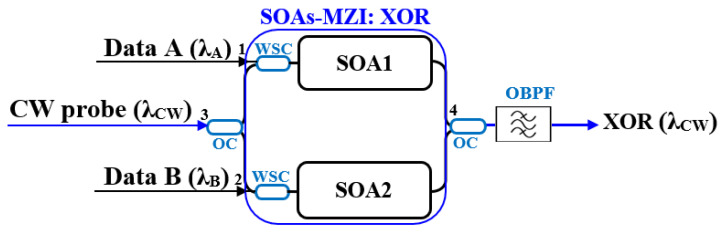
XOR operation with the SOAs-MZI schematic diagram. OBPF: optical bandpass filter [18], “reprinted with permission from Ref. [18], copyright (2022), Springer Nature”.

**Figure 7:**
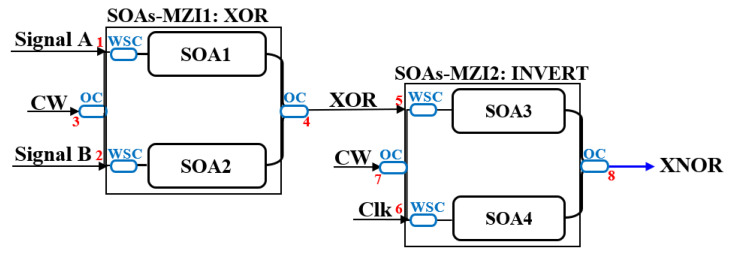
XNOR operation with the SOAs-MZI schematic diagram [38], “reprinted with permission from Ref. [38], copyright (2021), Elsevier”.

This section systematically presents the schematic diagrams of typical implementation schemes for basic all-optical logic gates (AND, NAND, OR, NOR, XOR, and XNOR), as shown in Figure 2, Figure 3, Figure 4, Figure 5, Figure 6, and Figure 7 [5,18,20,37,38]. Each figure will include the input signals A and B, CW probe, the core SOA nonlinear unit, and necessary signal processing units (such as interferometers, filters, couplers), as well as the optical path and logical relationship of the final output signal. The truth tables of the corresponding logic gates are shown in Table 1. The implementation principle and main implementation scheme of each logic gate will be introduced in Section 4.

## 3. Physical Mechanism and Evolution of SOAs

### 3.1. Conventional SOA

#### 3.1.1. Structure Characteristics

**Figure 8:**
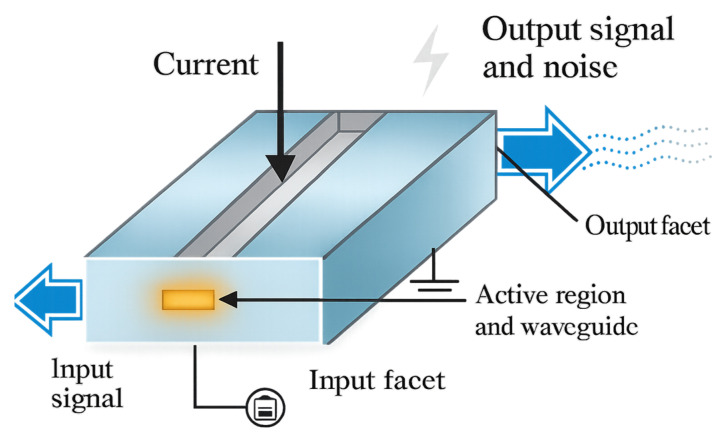
Schematic diagram of an SOA [5], “adapted from Ref. [5], World Scientific (2015)”.

Conventional bulk and quantum well SOA is the cornerstone of all-optical logic gate research, whose working principle is based on the particle number inversion and stimulated emission process achieved in the semiconductor active region (AR) under forward bias, as shown in Figure 8 [5]. This device is driven by current, where the AR transfers gain to the input signal via stimulated emission, with the output signal accompanied by noise. This superimposed noise is ASE, generated by the amplification process. The amplification principle is based on stimulated emission. An injected current causes the AR to achieve a particle number inversion, resulting in the phenomenon of conduction band electrons and valence band holes recombining. At the same time, photons with the same phase, polarization, and direction as the incident photons are emitted, thereby achieving coherent light amplification. The net power gain G can be expressed as follows [5]:

(5) $$G=exp\left[\left(\Gamma g\left(N\right)-\alpha_{int}\right)L\right],$$

Here, Γ is the light field limiting factor, $g\left(N\right)$ is the material gain coefficient related to the carrier concentration N, $\alpha_{int}$ is the internal loss, and L is the device length [49].

**Figure 9:**
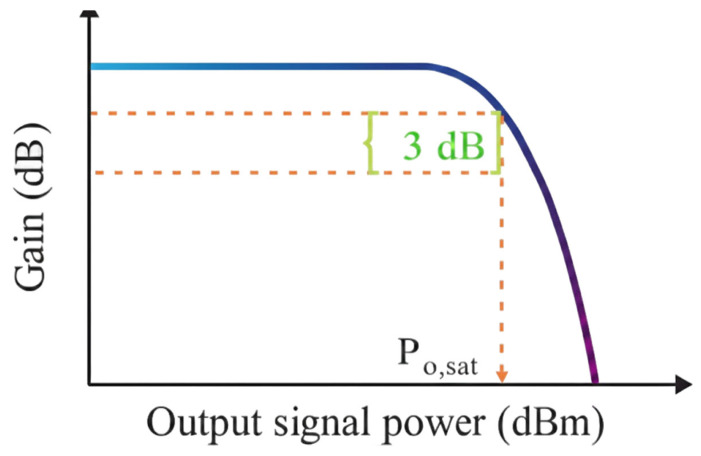
Typical SOA gain versus output signal power [5], “adapted from Ref. [5], World Scientific (2015)”.

The gain saturation characteristic is described by the saturated output power $P_{o,sat}$, as shown in Figure 9 [5]. As the input signal power increases, the gain also decreases, and this gain saturation can cause severe signal distortion. SOA is usually used to amplify modulated optical signals. If the signal power is high, the gain will saturate. If the gain dynamic change in the amplifier is a slow process, then this problem will not be very serious. SOA also exhibits nonlinear behavior. Based on the XPM, XGM, and FWM nonlinear characteristics in SOA, whose application fields include wavelength conversion, optical demultiplexing in high-speed transmission, optical logic devices, etc., it is the key to achieving high-quality output.

#### 3.1.2. Performance Bottleneck

The core bottleneck of conventional SOA is its slow gain recovery dynamics, with the recovery time $\tau_{c}$ typically ranging from 0.1 to 1 ns [50], which is dominated by processes such as spontaneous-emission lifetime and Auger recombination. This leads to a significant code type effect. From the single-mode rate equation, the following can be understood [5]:

(6) $$\frac{dN}{dt}=\frac{I}{qV}-\frac{N}{\tau_{c}}-\frac{\Gamma g\left(N\right)}{A\hbar \omega }P_{sig}\left(t\right)$$

Here, I represents the injected current, q is the electron charge, V is the volume of the AR, A is the area of the mode field, and $P_{sig}\left(t\right)$ is the power of the signal light. Here, ℏω denotes the photon energy, where ℏ is the reduced Planck constant and ω is the angular frequency of the optical signal. When consecutive “1” codes occur in the high-speed bit stream, the carriers are continuously consumed (the third term in the above equation), and they cannot fully recover within the bit period. This leads to a decrease in the gain experienced by subsequent “1” codes, which is known as the code type effect, causing waveform distortion and degradation of the extinction ratio. It is worth noting that the phase recovery due to the in-band relaxation process is usually faster than the gain recovery, but both together constitute the fundamental limitation of conventional SOAs in achieving ultra-high-speed all-optical logic [51]. Despite the existence of the above bottleneck, conventional SOAs served perfectly as a principal verification platform due to their mature fabrication process and high nonlinear coefficients in the early stage of technological development. The rate and code type effects exposed by them directly drove the evolution of subsequent devices.

### 3.2. QD-SOA

#### 3.2.1. Structure Characteristics

**Figure 10:**
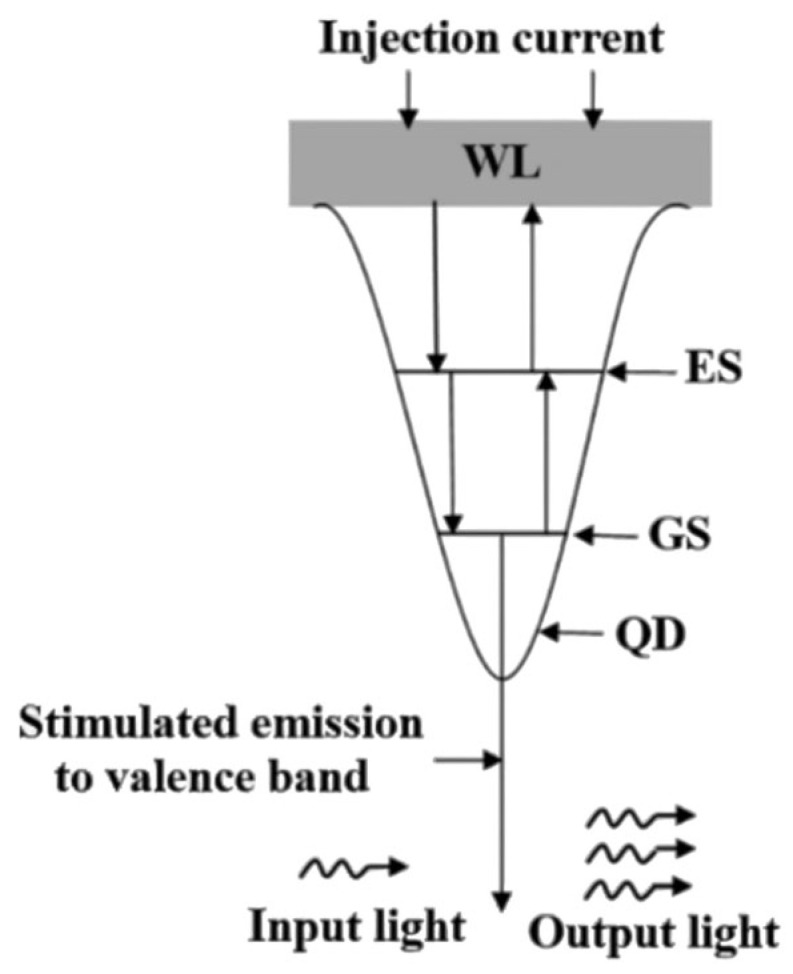
Energy level and carrier dynamics diagram of QD [53], “reprinted with permission from Ref. [53], copyright (2009), SPIE”.

The core physical advantage of QD-SOAs stems from the three-dimensional quantum confinement effect generated by the semiconductor nanostructures in their AR. This has become a key platform for breaking through the speed limitations of conventional SOA and achieving ultra-high-speed all-optical signal processing. This effect completely alters the electronic state properties of the material, which are different from the continuous energy bands of bulk or quantum wells. In a quantum dot, the carrier motion is restricted in all three spatial dimensions, forming a series of discrete energy levels, mainly including the ground state (GS) and excited state (ES) [52]. The state density function, under ideal conditions, approximates the superposition of a series of δ-functions, presenting discrete, atomic-like characteristics. This unique energy level structure is the fundamental physical basis for QD-SOA to achieve ultrafast carrier dynamics, extremely high differential gain, low noise coefficient, and relative insensitivity to temperature changes and polarization states. To achieve sufficient mode gain and meet the requirements of optical integration, the AR of practical QD-SOA usually adopts a multi-layer stacked structure. QD layers are generally self-assembled and grown on GaAs/InGaAs substrates through the Stranski–Krastanov (S-K) mode, such as growing InAs QDs on GaAs. This multi-layer design has multiple functions. Firstly, it significantly increases the overlap integral of the optical field with the AR, thereby enhancing the limiting factor Γ. Secondly, it helps to compensate for the non-uniform broadening caused by QD size and composition inhomogeneities, smoothing the gain spectrum. Finally, through engineering design, the polarization-dependent gain of the device can be reduced, enhancing its applicability in practical systems. More importantly, QDs together with the surrounding wetting layer (WL) form a complex carrier transport and relaxation system. The WL acts as a “distribution center” for carrier injection, where carriers are first injected into the continuous state of the WL, then are quickly captured to the ES of the quantum dots, and finally reach the GS through an extremely fast in-band relaxation process. This efficient relaxation path is the key mechanism for achieving picosecond-level ultrafast gain recovery, thereby supporting ultra-high-speed all-optical logic operations of 160 Gb/s and above, as shown in Figure 10 [53]. In summary, QD-SOA provides an ideal device platform for realizing the next-generation high-speed and high-efficiency all-optical signal processing through its discrete energy level structure, carefully designed multi-layer AR, and efficient carrier relaxation pathway.

#### 3.2.2. Dynamics Model

The core of ultrafast dynamics in QD-SOA stems from its discrete energy level structure, which can be modeled by a set of coupled rate equations. These equations model the dynamic transport and relaxation processes of carriers between WL, ES, and GS. This model is crucial for understanding the picosecond-scale gain recovery and suppression of code type effects [54,55]. The carrier density $N_{W}$ in the WL is as follows [5]:

(7) $$\frac{dN_{W}}{dt}=\frac{J}{ed_{W}}-\frac{N_{W}}{\tau_{W}^{nr}}-\frac{N_{W}\left(1-f_{ES}\right)}{\tau_{W\to ES}}+\frac{N_{ES}f_{ES}}{\tau_{ES\to W}}-R_{st,W},$$

Here, J represents the injection current density, e represents the electron charge, $d_{W}$ is the thickness of the wet layer, $\tau_{W}^{nr}$ is the non-radiative recombination lifetime in WL, $\tau_{W\to ES}$ is the carrier capture time from WL to ES (~1–10 ps), $\tau_{ES\to W}$ is the reverse thermal escape time, $f_{ES}$ is the carrier occupation probability of ES, and $R_{st,W}$ is the stimulated emission rate in WL. The ES carrier’s occupation probability is $f_{ES}$:

(8) $$\frac{{df}_{ES}}{dt}=\frac{N_{W}\left(1-f_{ES}\right)}{N_{Q}d_{W}\tau_{W\to ES}}-\frac{f_{ES}}{\tau_{ES}^{sp}}-\frac{N_{ES}\left(1-f_{GS}\right)}{\tau_{ES\to GS}}+\frac{f_{GS}\left(1-f_{ES}\right)}{\tau_{GS\to ES}}-\frac{\Gamma v_{g}g_{ES}}{N_{Q}}S\left(2f_{ES}-1\right),$$

Here, $N_{Q}$ is the surface density of quantum dots, $\tau_{ES}^{sp}$ is the spontaneous-emission lifetime of ES, and $\tau_{ES\to GS}$ is the ultrafast in-band relaxation time from ES to GS, which can be as short as the sub-picosecond to picosecond level, being the core of the ultrafast dynamics of QD-SOA. $\tau_{GS\to ES}$ is the reverse thermal excitation time, $v_{g}$ is the group velocity, $g_{ES}$ is the differential gain of ES, S is the photon density, and Γ is the light field confinement factor. The expression for the GS carrier’s occupation probability is $f_{GS}$:

(9) $$\frac{df_{GS}}{dt}=\frac{f_{ES}\left(1-f_{GS}\right)}{\tau_{ES\to GS}}-\frac{f_{GS}}{\tau_{GS}^{sp}}--\frac{f_{GS}\left(1-f_{ES}\right)}{\tau_{GS\to ES}}-\frac{\Gamma v_{g}g_{GS}}{N_{Q}}S\left(2f_{GS}-1\right),$$

The last term of the formula represents stimulated emission, which directly consumes GS carriers and generates signal gain. The recovery speed of GS is determined by $\tau_{ES\to GS}$ and the supply speed of carriers from WL to ES. At high-speed modulation, in addition to the average carrier dynamics, ultrafast nonlinear effects are crucial. The gain coefficient $g\left(t\right)$ of QD-SOA is determined by carrier density dynamics and nonlinear effects (CH and SHB), and its expression is as follows:

(10) $$g\left(t\right)=\Gamma \left[g_{GS}N_{Q}\left(2f_{GS}\left(t\right)-1\right)+g_{ES}N_{Q}\left(2f_{ES}\left(t\right)-1\right)\right]-\varepsilon_{SHB}S\left(t\right)-\varepsilon_{CH}\int_{-\infty }^{t}h_{CH}\left(t-t^{\prime }\right)S\left(t^{\prime }\right)dt^{\prime },$$

Here, $\varepsilon_{SHB}$ and $\varepsilon_{CH}$ are the nonlinear gain compression factors that characterize the intensity of SHB and CH effects [56]. SHB originates from the localized consumption of GS carriers by the signal light at a specific frequency within the gain spectrum, while CH stems from the heating of the carrier distribution to a higher energy, resulting in the overall redshift and distortion of the gain spectrum. The response function $h_{CH}\left(t\right)$ typically has a time constant of picosecond order. The nonlinear phase shift $\phi_{NL}\left(t\right)$ coupled with the gain dynamics is dominated by the linewidth enhancement factor α. In QD-SOA, it is usually necessary to distinguish the α factor related to different processes:

(11) $$\phi_{NL}\left(t\right)\approx -0.5\;\left[\alpha_{GS}\Delta g_{GS}(t)+\alpha_{CH}{\Delta g}_{CH}(t)]L\right],$$

Here, $\alpha_{GS}$ corresponds to the phase–amplitude coupling of interband transitions, while $\alpha_{CH}$ corresponds to the stronger phase modulation caused by the CH process [57]. The relatively low $\alpha_{GS}$ value of QD-SOA implies that the signal chirp caused by XPM is smaller, which is beneficial for cascading. However, the larger $\alpha_{CH}$ will introduce significant instantaneous phase nonlinearity under ultrashort pulses, and this needs to be considered in the design of the interferometer [58].

#### 3.2.3. Performance Advantage

The root cause of ultrafast gain recovery (reaching picosecond to femtosecond levels) lies in the fact that the ES carriers can serve as a rapid replenishment source for the GS carriers. When the GS carriers are consumed by the signal light, the ES carriers are replenished through extremely rapid in-band scattering, thereby fundamentally suppressing the code-type effect from a physical mechanism. The excellent characteristics derived from this have comprehensively enhanced the logic performance. The wide-gain-spectrum and low-dispersion characteristics reduce the stringent requirements for the working wavelength, and the strong localization of the carriers makes them extremely insensitive to temperature changes. Meanwhile, QD-SOA typically exhibits a lower linewidth enhancement factor, which means a weaker phase–amplitude coupling, which is beneficial for signal fidelity and cascading. However, its development still faces significant challenges, such as non-uniformity of quantum dot sizes leading to non-uniform broadening of the gain spectrum, complex gain saturation characteristics, and the need for major process challenges in achieving high-density and high-quality quantum dot epitaxial growth.

### 3.3. PhC-SOA

#### 3.3.1. Structure Characteristic

**Figure 11:**
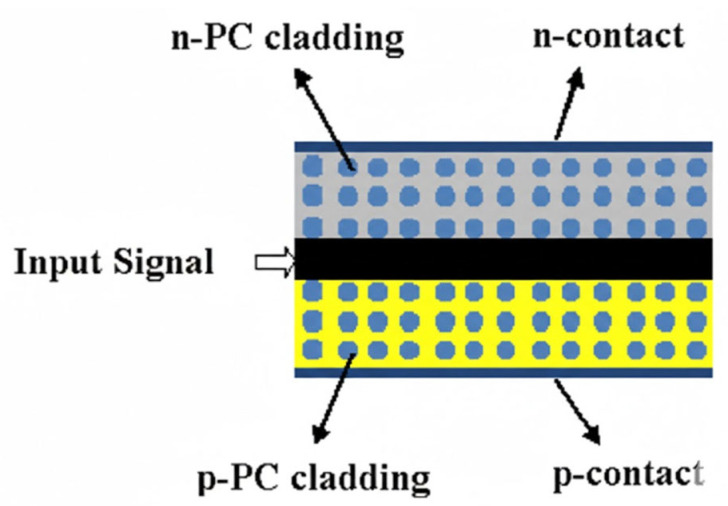
Schematic diagram of PC-SOA’s waveguide [59], “reprinted with permission from Ref. [59], copyright (2015), Elsevier”.

A photonic crystal is an artificial microstructure with a periodically distributed dielectric constant, and its core physical property lies in generating a photonic bandgap, meaning that light within a specific frequency range cannot propagate within it. By introducing point defects or line defects, it is possible to achieve local guidance and manipulation of photons. By combining photonic crystal waveguides with the AR of a semiconductor optical amplifier, a photonic crystal semiconductor optical amplifier is formed. The design aims to utilize the unique optical properties of photonic crystals to break through the performance limitations of conventional SOA [59]. A typical PhC-SOA is based on the III-V material system, such as InP/InGaAsP, and adopts a two-dimensional triangular-lattice photonic crystal plate structure, as shown in Figure 11 [59]. Periodic arrays of air holes are etched in the semiconductor plate, and by introducing a line defect with a missing air hole, a channel that can guide light is formed. This channel is the effective active waveguide of the device. By precisely designing the lattice constant (a), the radius of the air holes (r), and the thickness of the plate, the position and width of the photonic bandgap can be controlled near the target wavelength (e.g., 1.55 μm communication window), and the line defect waveguide can support the propagation of slow-light modes, enabling high-speed all-optical logic gate operations [60].

The slow-light effect is the core feature of PhC-SOA, and its quantitative description is a significant increase in the group refractive index $n_{g}$:

(12) $$n_{g}=\frac{c}{v_{g}}=n_{eff}+\omega \frac{dn_{eff}}{d\omega },$$

Here, c represents the speed of vacuum light, $v_{g}$ is the group velocity, $n_{eff}$ is the effective refractive index of the mode, and ω is the angular frequency. Near the band edges of the photonic crystal waveguide, due to intense dispersion (with a large value of $dn_{eff}/d_{\omega }$, $n_{g}$ can reach 50–100, which is more than 3–4 times higher than that of traditional strip waveguides [59,61]. The introduced slow-light factor $S=n_{g}/n_{g0}$ (where $n_{g0}$ is the reference group refractive index) is a key indicator for measuring the degree of light speed reduction [62,63]. The extremely high group refractive index means that when photons pass through the same physical length L of the device, their effective interaction time $\tau_{eff}=n_{g}L/c$ is greatly prolonged. This brings two fundamental advantages: one is that the required physical device length L for achieving a given net gain $G=\exp\left(\Gamma gL\right)$ can be significantly shortened, thus laying the foundation for the realization of extremely high-density photonic integrated circuits (PICs). The other is the synchronous increase in the energy density of the light field and the action time, creating conditions for the significant enhancement of nonlinear effects. However, this strong dispersion characteristic is accompanied by a narrow slow-light optical bandwidth, limiting the signal spectrum width it can handle and imposing stringent requirements of nanometer-level manufacturing precision.

#### 3.3.2. Dynamics Model

The ultrafast nonlinear dynamics of PhC-SOA are pivotal for achieving high-performance all-optical logic. Its unique slow-light effect not only reduces the size of the device but also fundamentally enhances the intensity of the interaction between light and matter. The slow-light factor serves as the core parameter quantifying this enhancement. Slow light also brings about an increase in the energy density of the light field and an extension of the effective interaction time. Therefore, the nonlinear phase shift $\Delta \emptyset_{Nl}$ caused by the carrier effect is doubly enhanced [64,65,66]:

(13) $$\Delta \emptyset_{NL}^{PhC}\propto \gamma \left(S\cdot P\right)L\cdot S\approx \gamma PL\cdot S^{2},$$

Here, γ is the nonlinear coefficient, P is the input power, and L is the physical length [67]. This implies that to achieve the same nonlinear phase shift, the pump power or the device length required by PhC-SOA can theoretically be reduced by $S^{2}$ times. This points toward a disruptive path to all-optical logic operations with extremely low power consumption and ultra-compact dimensions. To describe its response under ultrashort pulses, the dynamic of PhC-SOA typically employs a multicomponent gain model. The total integrated gain $h\left(t\right)$ is decomposed into the sum of multiple dynamic components [66]:

(14) $$h\left(t\right)=h_{CD}\left(t\right)+h_{CH}\left(t\right)+h_{SHB}\left(t\right)+h_{PC}\left(t\right),$$

The dynamic equations for each item are as follows: Firstly, $h_{CD}\left(t\right)$ originates from the slow gain component of carrier depletion and recombination. The time constant $\tau_{c}$ is of the nanosecond order and is the main nonlinear source of the conventional SOA:

(15) $$\frac{dh_{CD}\left(t\right)}{dt}=\frac{h_{0}-h_{CD}\left(t\right)}{\tau_{c}}-\frac{P_{in}\left(t\right)}{E_{sat}}\left(e^{h\left(t\right)}-1\right),$$

$h_{CH}\left(t\right)$ and $h_{SHB}\left(t\right)$ are the CH and SHB components, respectively, belonging to ultrafast in-band nonlinear effects. The time constants $\tau_{CH}$ and $\tau_{SHB}$ are in the picosecond to femtosecond range, respectively, resulting in instantaneous gain compression and saturation related to the carrier change rate. Their expressions are, respectively, as follows:

(16) $$\frac{dh_{CH}\left(t\right)}{dt}=-\frac{h_{CH}\left(t\right)}{\tau_{cH}}-\varepsilon_{CH}\frac{P_{in}\left(t\right)}{E_{sat}}\left(e^{h\left(t\right)}-1\right),$$

(17) $$\frac{dh_{SHB}\left(t\right)}{dt}=-\frac{h_{SHB}\left(t\right)}{\tau_{SHB}}-\varepsilon_{SHB},$$

$h_{PC}\left(t\right)$ is the additional nonlinear dispersion and absorption modulation caused by slow light, as well as the unique dynamics introduced by the boundary effect of photonic crystals, which is directly related to the input optical power [59]. Its expression is as follows:

(18) $$\frac{dh_{PC}\left(t\right)}{dt}=-\frac{h_{PC}\left(t\right)}{\tau_{pc}}-\kappa_{PC}\frac{P_{in}\left(t\right)}{E_{sat}},$$

Here, the coefficient $\kappa_{PC}$ reflects the contribution of the slow-light effect of the photonic crystal to the nonlinear enhancement. $P_{in}\left(t\right)$ represents the instantaneous input optical power, $E_{sat}$ is the saturation energy, and R is the background loss. The nonlinear phase change $\phi_{NL}\left(t\right)$ coupled with the gain dynamics is mainly dominated by the α (linewidth enhancement factor), and its expression is as follows:

(19) $$\phi_{NL}\left(t\right)=-0.5\;\left[\alpha_{CD}{\;h}_{CD}\left(t\right)+\alpha_{CH}\;h_{CH}\left(t\right)+\alpha_{PC}\;h_{PC}\left(t\right)\right],$$

Here, $\alpha_{CD}$ is the traditional interband transition linewidth enhancement factor, while $\alpha_{CH}$ corresponds to the equivalent linewidth enhancement factor for the carrier heating process, and $\alpha_{PC}$ characterizes the additional phase modulation efficiency caused by the photonic crystal structure [68]. This model indicates that PhC-SOA not only inherits the nonlinear mechanisms such as CD, CH, and SHB in conventional SOA and QD-SOA, but also introduces the unique nonlinear term ($h_{PC}$) specific to the photonic crystal structure. This enables further engineering regulation of its ultrafast response and phase modulation capabilities, providing a new physical degree of freedom for achieving ultra-high-speed and low-power all-optical logic gates.

#### 3.3.3. Performance Advantage

This slow-light effect brings about a revolutionary nonlinear enhancement. Based on the $S^{2}$ enhancement of nonlinear efficiency, it is expected to achieve sub-milliwatt-level switching power consumption and micrometer-level functional units. This means that for the same nonlinear phase shift, the required device length or pump power can be reduced by a square inverse order of magnitude. Therefore, PhC-SOA theoretically represents a disruptive path towards ultra-low power consumption and extremely compact size all-optical logic operations. However, its inherent challenges are also significant. The slow-light effect is accompanied by an extremely narrow optical bandwidth, limiting the signal spectrum that can be processed. Manufacturing defects of nano-scale structures will introduce severe scattering losses. Changes in the slow-light mode distribution also bring about mode-matching problems with traditional waveguides. The performance of PhC-SOA is highly dependent on the sub-wavelength scale processing accuracy. It represents a frontier direction of using the spatial structure of the optical field to regulate, rather than simply material modification, to break through the limit of nonlinear efficiency.

### 3.4. RSOA

#### 3.4.1. Structure Characteristic

**Figure 12:**
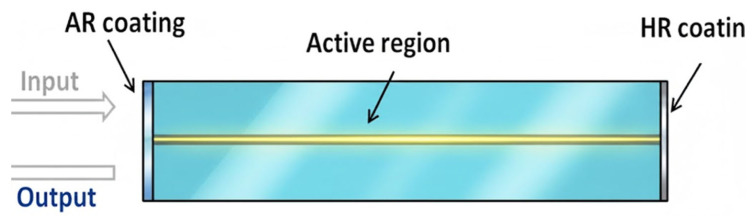
Schematic of RSOA device [5], “adapted from Ref. [5], World Scientific (2015)”.

The RSOA is a device structure specifically optimized for the high-density and high-function integration requirements of PICs. The core AR material system of RSOA is like that of conventional SOA, but its core structure innovation lies in the asymmetric cavity surface reflection rate design, as shown in Figure 12 [5]. Unlike traditional traveling wave SOAs, which have anti-reflection coatings (AR-coatings) on both ends to suppress Fabry–Pérot resonance, RSOAs usually have a high-reflection facet (HR) coating at one end (the rear surface) and an anti-reflection film or low-reflection facet coating at the other end (the front surface). The AR-coating at the front surface (the signal input/output end) reduces the reflection rate to below 0.1% to maximize the coupling of the incoming/outgoing optical signals and suppress Fabry–Perot resonance. The rear surface is coated with an HR facet (reflection rate > 90%), forming an optical cavity mirror.

This design establishes a unidirectional optical path, where the incident optical signal enters the AR through the AR-coating facet, undergoes stimulated emission gain and depletes carriers during the forward propagation. Upon reaching the HR facet, the majority of the light is reflected and travels back along the original path, passing through the AR-coated facet again to experience a second amplification gain, and finally exits from the same AR-coating facet [69]. This structure brings two fundamental advantages. Firstly, the port number is halved, making it naturally suitable for “U-shaped” or folded optical paths in PICs layout, significantly saving chip area and enhancing integration friendliness. Secondly, the bidirectional amplification mechanism approximately doubles the effective gain length of the signal light, thereby obtaining sufficient gain in a shorter physical cavity length (which can be shortened to several hundred micrometers) and significantly enhancing nonlinear effects due to the prolonged interaction time between light and matter. However, this resonant structure also causes uneven carrier density distribution in space and introduces end-face feedback, whose dynamic behavior is more complex than that of traditional traveling wave SOA.

#### 3.4.2. Dynamics Model

To efficiently simulate the complex dynamic behavior of RSOA, it is necessary to incorporate the effects of the round-trip effect and end-face feedback in the conventional SOA multicomponent model through an equivalent approach. Usually, an extended lumped parameter model is adopted, which decomposes the dynamic response of the total gain $h\left(t\right)=\ln\sqrt{G(t)/R_{HR}}$ (where $G\left(t\right)$ is the single-path power gain and $R_{HR}$ is the power reflection coefficient at the rear end face) into the contributions of multiple physical processes [70]:

(20) $$h\left(t\right)=h_{CD}\left(t\right)+h_{CH}\left(t\right)+h_{SHB}\left(t\right),$$

The equations for each component are as follows:

(21) $$\frac{dh_{CD}\left(t\right)}{dt}=\frac{h_{0}-h_{CD}\left(t\right)}{\tau_{c}}-\frac{P_{in}\left(t\right)}{E_{sat}}\left[e^{2h\left(t\right)}-1\right]-R_{eff},$$

(22) $$\frac{dh_{CH}\left(t\right)}{dt}=-\frac{h_{CH}\left(t\right)}{\tau_{cH}}-\varepsilon_{CH}\frac{P_{in}\left(t\right)}{E_{sat}}\left[e^{2h\left(t\right)}-1\right],$$

(23) $$\frac{dh_{SHB}\left(t\right)}{dt}=-\frac{h_{SHB}\left(t\right)}{\tau_{SHB}}-\varepsilon_{SHB}\frac{dh_{CD}\left(t\right)}{dt},$$

Here, the key difference from the single-path model lies in the fact that the gain factor in the dual-layer gain saturation term is $e^{2h\left(t\right)}$, rather than $e^{h\left(t\right)}$ in the single-path model. This precisely reflects the total gain experienced by the light during the round-trip double path, resulting in a more significant saturation characteristic of RSOA, that is, it is more likely to enter the gain saturation state, and thus, the establishment efficiency of the nonlinear phase shift is higher. The effective feedback term $R_{eff}$ integrates the effective optical feedback introduced by the HR end-face reflection and the influence of the internal loss $\alpha_{loss}$ in the AR ($R_{eff}\propto {\left(1-R_{HR}e^{-2\alpha_{loss}L}\right)}^{-1}$), which corrects the small signal gain and affects the stable state. $h_{CH}\left(t\right)$ and $h_{SHB}\left(t\right)$ correspond to the carrier heating and spectral hole effects respectively, and their time constants $\tau_{cH}$ and $\tau_{SHB}$ are in the range of sub-picoseconds to picoseconds, determining the response speed of RSOA to ultrashort pulses. Based on the above gain dynamics, the total output power gain $G_{total}\left(t\right)$ and nonlinear phase shift $\emptyset_{NL}\left(t\right)$ of RSOA can be expressed as follows:

(24) $$G_{total}\left(t\right)=R_{HR}\exp\left[2\left(h\left(t\right)-\alpha_{loss}L\right)\right],$$

(25) $$\emptyset_{NL}\left(t\right)=-0.5\;\left[\alpha h_{CD}\left(t\right)+\alpha_{CH}h_{CH}\left(t\right)\right],$$

In the gain formula, a factor of 2 once again demonstrates the dual-path effect, and $R_{HR}$ is the key parameter determining the final output power. The nonlinear phase shift equation indicates that phase modulation mainly originates from the carrier depletion and carrier heating processes and is modulated, respectively, by α (linewidth enhancement factor) and the carrier heating line width enhancement factor $\alpha_{CH}$ (carrier heating line width enhancement factor). Due to the stronger interaction between the optical field and the carriers in RSOA, its effective nonlinear coefficient is usually higher than that of conventional SOA, enabling interference-type logic gates based on RSOA to achieve the required π phase shift at lower input power or shorter device length, which is of great significance for reducing power consumption and achieving compact PICs integration.

#### 3.4.3. Performance Advantage

The RSOA achieves a unidirectional and bidirectional amplification optical path architecture through its unique asymmetric cavity surface design, namely, AR-coating at the front end and HR at the rear end. This fundamental structural innovation makes it an ideal core component for PICs. Its single-port input/output characteristic is naturally compatible with the “U-shaped” folding layout in planar optical paths, significantly enhancing the space utilization efficiency and integration friendliness. Owing to the effective doubling of the effective action length achieved by the light traveling back and forth in the cavity once, RSOA can provide higher gain efficiency in a shorter physical size and significantly enhance the interaction between light and carriers, thereby achieving a better effective nonlinear coefficient than traditional traveling wave SOA. This enables interferometer-based RSOA to achieve the required nonlinear phase shift with lower power consumption or a more compact size, providing a critical nonlinear source and gain foundation for high-density integrated all-optical signal processing. Therefore, RSOA transcends the role of traditional discrete components, serving as a strategic “gain interface” and “nonlinear engine” that bridges low loss passive waveguide platform with complex active processing functions. It is a key enabling device for promoting all-optical logic from discrete demonstrations to large-scale, multifunctional chip integration.

### 3.5. CR-SOA

#### 3.5.1. Structure Characteristic

**Figure 13:**
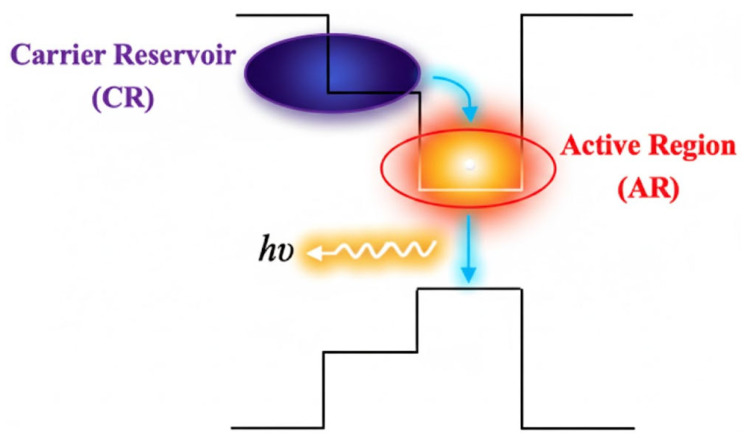
Band diagram of CR-SOA [5], “adapted from Ref. [5], World Scientific (2015)”.

CR-SOA is a device that innovates the structure of conventional SOA through band engineering, aiming to solve the bottleneck problem of slow gain recovery in the conventional SOA structure. The typical structure of CR-SOA is to integrate an additional carrier reservoir (CR) layer on one or both sides of the traditional AR through a thin barrier layer. The core physical idea of this design is to construct a “water reservoir” of carriers, as shown in Figure 13 [5]. To achieve effective “storage” and “rapid supply” functions, the storage layer is usually designed to have a wider bandgap than AR. The thickness and height of the barrier layer between the AR and the storage layer have been carefully optimized to meet two seemingly contradictory but crucial requirements. One is that it should be thin enough or the barrier low enough to ensure that carriers can cross quickly when needed. The other is that under static bias, it should be able to effectively confine most of the injected carriers within the storage layer rather than in the AR [71]. Under electric pumping, carriers (electrons and holes) are preferentially injected and stored in the wide-bandgap storage layer, forming a high concentration CR [72]. When a signal light pulse carrying the logic “1” is injected into the AR, it rapidly consumes the carriers in the AR through stimulated emission, resulting in local gain saturation [73]. At this point, the quasi-Fermi level difference between the storage layer and AR rapidly increases. This drives the pre-stored high concentration carriers in the storage layer to be injected into the AR through tunneling or thermal emission mechanisms at an extremely fast speed (0.5–10 ps), quickly replenishing the consumed carriers and thereby achieving ultrafast recovery of macroscopic gain and nonlinear phase shift [74].

CR-SOA typically employs multi-quantum well or bulk heterostructures based on InP/GaAs materials to achieve the band design. Its core advantage lies in design flexibility, where the materials, thickness, and doping concentration of AR, storage layer, and barrier layer can all be independently controlled [74]. This provides additional flexibility for optimizing the performance of the device under specific targets (such as a specific recovery speed, saturated output power, working bandwidth). Functionally, the “storage-supply” mechanism of CR-SOA is like the carrier supply pathway of “WL-quantum dots” in QD-SOA in terms of the physical Mechanism. However, CR-SOA is engineered at a more macroscopic energy band structure level and theoretically has stronger controllability and predictability.

#### 3.5.2. Dynamics Model

The dynamic behavior of CR-SOA lies at the core of its dual-region carrier exchange system. To accurately predict its response at rates ranging from tens to hundreds of Gb/s, its model needs to simultaneously describe the carrier transport between CR and AR, as well as the ultrafast in-band nonlinear effects. The carrier dynamics coupled equation system can be expressed as follows [75]:

(26) $$\frac{dN_{CR}}{dt}=\frac{I}{qV_{CR}}-\frac{N_{CR}}{\tau_{cap}}-R_{sp,CR}\left(N_{CR}\right),$$

(27) $$\frac{dN_{AR}}{dt}=\frac{N_{CR}}{\tau_{cap}}-R_{sp,AR}\left(N_{AR}\right)-\frac{\Gamma g\left(N_{AR}\right)}{\hbar \omega A}P_{sig}\left(t\right),$$

Here, $N_{CR}$ and $N_{AR}$ represent the carrier densities of the CR layer and the AR layer respectively, I is the injection current, and $V_{CR}$ is the storage volume. $\tau_{cap}$ is the characteristic time for carriers to be captured from the CR layer to the AR layer, which determines the speed of ultrafast replenishment and is usually designed at the picosecond level (1–10 ps). $R_{sp}$ represents the spontaneous emission rate of CR and AR, $P_{sig}(t)$ is the power of the signal light, Γ is the limiting factor, g is the gain coefficient, and A is the area of the moire field. The total effective integral gain h(t) can be decomposed into the sum of contributions from multiple dynamic processes, and its model can be expressed as follows:

(28) $$h\left(t\right)=h_{0}+h_{CR}\left(t\right)+h_{CH}\left(t\right)+h_{SHB}(t),$$

Here, $h_{0}$ represents the small signal integral gain associated with the bias current. The $h_{CR}\left(t\right)$ directly reflects the contribution of ultrafast carrier replenishment in the CR layer to the recovery of AR gain, with its dynamics governed by $\tau_{cap}$. $h_{CH}\left(t\right)$ and $h_{SHB}(t)$ represent gain compression induced by two ultrafast in-band nonlinear effects, CH and SHB, respectively. Their characteristic recovery time constants ($\tau_{CH},\tau_{SHB}$) typically range from sub-picoseconds to several picoseconds. The dynamics of these ultrafast components are typically described by first-order differential equations, whose form reflects their excitation by the signal light followed by subsequent relaxation:

(29) $$\frac{dh_{i}}{dt}=-\frac{h_{i}}{\tau_{i}}-\varepsilon_{i}\frac{P_{in}\left(t\right)}{P_{sat}}\left(e^{h\left(t\right)}-1\right)\;\;\;i\in \left\{CR,CH,SHB\right\},$$

In the formula, $\varepsilon_{i}$ represents the nonlinear gain suppression factor of each effect, $P_{in}\left(t\right)$ is the input optical power, and $P_{sat}$ is the saturation power.

#### 3.5.3. Performance Advantage

Benefiting from the ultrafast carrier supply of the CR layer, CR-SOA exhibits significantly dynamic characteristics compared to conventional SOA. Following strong optical pulse perturbations, its gain and phase recovery present typical double-exponential time scale characteristics. One is a rapid initial-recovery stage dominated by the CR layer and the CH/SHB effect, with a time constant of several ps. Second is a slow complete-recovery stage determined by the interband recombination lifetime of the AR layer, with a time constant of 10~100 ps. For high-speed logic operations, the rapid initial-recovery stage is crucial for reconstructing sufficient gain difference within the bit time slot, thereby suppressing the code type effect. Therefore, the essence of the performance improvement of CR-SOA lies in that it does not change the intrinsic carrier recombination lifetime of the AR material, but in introducing a carrier “reservoir” that optimizes carrier distribution and scheduling processes across both spatial (additional carrier sources) and temporal (ultrafast supply channels) dimensions. It transforms the recovery process from relying on the slow “generation-combination” cycle to an efficient “scheduling-supplement” mode, thereby achieving a faster effective recovery speed at the system level. Combined with its higher manufacturing maturity and design flexibility compared to the QD process, CR-SOA becomes an attractive engineering optimization paradigm for all-optical logic gate applications in the 40 Gb/s to 100 Gb/s rate range, balancing performance, complexity, and cost. It is an important transitional technology from conventional SOA to the more complex material system during the evolution process.

## 4. Applications and Performance of All-Optical Logic Gates

### 4.1. Implementation of AND Gate

#### 4.1.1. Operation Principle

The implementation of the all-optical AND gate mainly relies on the nonlinear effects, such as XGM, XPM, and FWM, of the SOA, combined with different interferometer structures, to achieve high-speed AND logic operations, as shown in Figure 2 [20]. First, data A ($\lambda_{A}$) and its temporally delayed replica are injected into two SOAs via a WSC, respectively. At the same time, the data B ($\lambda_{B}$) is fed into both SOA1 and SOA2 through a 3 dB OC. When B = “0”, no light power centered at $\lambda_{B}$ is injected to configure the operation, resulting in no output at port 4. When B = “1” and A = “0”, the split portions of data B experience the same gain and phase shift in the two SOAs, and the phase vectors from the two paths cannot produce constructive interference, thus yielding a logical “0” at port 4. When B = A = “1”, the two branches of data B undergo different gain and XPM phase shifts in their respective SOAs, leading to constructive interference of the two beams at the output coupler and producing a logical “1” output. In the logic gate configuration, if the maximum conversion occurs at the output port when the phase difference between the two arms equals π. Consequently, the MZI generates a “1” at port 4 only when both data A and B are “1”, that is, the logical operation A AND B. Its truth table is shown in Table 1a.

#### 4.1.2. AND Logic Gate Technological Development-Based SOAs

Based on conventional bulk and quantum well SOA, the XGM scheme based on a single SOA or the XPM scheme in SOA-MZI are mostly adopted [76,77]. The technology is mature, but due to the long carrier recovery time, the rate and ER are both low. One study [78] experimentally demonstrated the all-optical AND gate operation based on the SOA-MZI differential scheme at a rate of 80 Gb/s and verified its functionality through numerical simulation. One study [79] numerically simulated a soliton all-optical AND gate at a rate of 80 Gb/s, and analyzed the influence of soliton characteristics and SOA parameters on the performance of the AND gate, indicating that solitons can improve the robustness and transmission distance of the signal. Subsequently, a new architecture, Turbo-Switched MZI (TS-MZI), was proposed, which further improved the operation rate and performance by optimizing the switching characteristics of the MZI. At a rate of 160 Gb/s, the study in [80] utilized TS-MZI to achieve error-free all-optical AND gate operation, with a QF 2.7 dB higher than the traditional design. One study [81] further proposed and analyzed the improved differential scheme combined with TS-MZI, achieving error-free all-optical AND gate operation and wavelength conversion at a rate of 640 Gb/s, which is the fastest AND gate based on bulk material SOA. Based on the QD-SOA scheme, it mainly utilizes its ultrafast carrier dynamics and implements high-speed AND operations in the QD-SOA-MZI structure, with a rate covering 160 Gb/s to over 1 Tb/s, and the ER is significantly improved to 12–20 dB, demonstrating advantages in high-speed and low-power processing. The researchers based on the InAs/GaAs QD-SOA scheme predicted that it has a potential working speed of 250 Gb/s [53]. Subsequently, all-optical AND gate based on QD-SOA-MZI was numerically simulated at a rate of 250 Gb/s, and the influence of ASE was taken into account, verifying the high-speed potential of QD-SOA [82]. One study [83] numerically studied and verified the QDSOA-TS-MZI to achieve all-optical XOR and AND logic gates at a rate of 1 Tb/s, further demonstrating the superiority of QD-SOA in ultra-high-speed logic operations. Another study [84] theoretically studied the application of two-photon absorption (TPA) in the QD-SOA all-optical AND gate, with a working rate of 2 Tb/s, proving that TPA can significantly improve the QF. Based on PhC-SOA, it mainly utilizes the slow-light effect to enhance nonlinearity. A further study [85] designs and simulates the all-optical AND logic gate in PhC-SOA with an ideal optical bandpass filter using FWM technology. In the proposed scheme, the required logical functions can be achieved without using continuous wave signals. The structure based on RSOA, due to its compact and easy-to-integrate characteristics, is mostly implemented using the integrated MZI/DI scheme to achieve a logic AND gate. The study in [86] theoretically studied the performance of the first all-optical logic AND gate implemented using RSOA as a nonlinear device at a rate of 120 Gb/s and experimentally proved that the performance is superior to conventional SOA at the target rate. Based on the CR-SOA structure, its special carrier storage layer is used to enhance the nonlinear effects, and is mostly used in integrated MZI structures, maintaining a high ER and good noise characteristics at a rate of 80–160 Gb/s. The TPA-enhanced CR-SOA-MZI scheme for the AND gate achieved a significantly higher speed of 320 Gb/s compared to the baseline 100 Gb/s, while simultaneously improving the QF from 14 to 14.82 [73], demonstrating a rare and advantageous performance scaling where both speed and signal integrity are enhanced.

### 4.2. Implementation of NAND Gate

#### 4.2.1. Operation Principle

The NAND gate is the logical complement of the AND gate. Its physical structure can be identical to the corresponding AND gate (especially in interferometer-based schemes). Therefore, realizing high-performance basic gates is a prerequisite for obtaining their complementary gates. The INVERT gate can be implemented as a cascaded combination to form a NAND gate, as shown in Figure 3 [5]. Based on the implementation of the AND gate, the amplifier boosts the output data to an appropriate power level and directs it into port 5, which serves as the data signal fed into the second CR-SOA-MZI2. One arm of this MZI is configured to perform the logical inversion. A clock pulse sequence with the same pulse shape and energy ($\lambda_{B}$) is injected into port 6 as a probe signal, while a CW probe is injected into port 7 as a control beam. Consequently, the output from port 8 corresponds to the modulo 2 additions of the data patterns of the signals injected into port 6 and port 5, that is, INVERT (A AND B), achieving A NAND B [87]. The corresponding truth table is shown in Table 1b.

#### 4.2.2. NAND Logic Gate Technological Development-Based SOAs

Conventional bulk and quantum well SOA typically employ cascading schemes based on XGM or differential phase modulation schemes for NAND functionality. Their advantages lie in mature technology and relatively simple structure, but are limited by carrier recovery time, with the rate typically ranging from 10 to 40 Gb/s, with a generally low ER (about 6–10 dB), and significant power consumption and code type effects. One study [88] adopted the classic SOA-MZI structure combined with XGM to achieve NAND functionality at a 10 Gb/s rate. Solutions for soliton all-optical NAND gates based on SOA-assisted MZI achieved a high QF at 80 Gb/s [89]. At the same rate, based on a single SOA cascaded DI, demonstrating significantly better performance compared to the cascaded MZI structure, with a QF reaching 10.75 [90]. The SOA-MZI structure based on the TPA effect can significantly increase the working rate to 250 Gb/s [91,92,93]. QD-SOA, with its ultrafast carrier dynamics and high nonlinear efficiency, is one of the optimal choices for achieving high-speed NAND gates. Experiments have proved that it can operate stably at rates ranging from 160 Gb/s to 320 Gb/s, with an ER of up to 12–18 dB, and has good tolerance to polarization and temperature fluctuations, making it a core device for future ultra-high-speed all-optical processing [94]. PhC-SOA utilizes the slow-light effect to greatly enhance the interaction between light and matter, making it possible to implement NAND gates at extremely low power consumption. Its schemes, based on XGM/XPM in photonic crystal waveguides or microcavities, although limited by bandwidth, can achieve extremely high extinction ratios and extremely low energy consumption, and have broad prospects in high-density, low-power photonic integrated circuits [95,96]. RSOA, with its compact, low-cost, and easy-to-integrate characteristics, is particularly suitable for scenarios with less stringent rate requirements but high integration and low cost, such as optical access networks and on-chip reconfigurable optical paths. Reference [97] used numerical simulation based on RSOA integration of MZI structure to implement a 120 Gb/s NAND gate, with performance superior to conventional SOA, especially considering ASE and harsh environmental conditions. CR-SOA effectively improves the speed and uniformity of nonlinear response through its unique carrier supply mechanism. The integration of MZI structure based on CR-SOA can achieve a superior ER and signal quality to conventional SOA at medium-high rates of 80–160 Gb/s, especially in suppressing code type effects [87]. In this structure, the use of the TPA effect enables a rate of up to 320 Gb/s [98].

### 4.3. Implementation of OR Gate

#### 4.3.1. Operation Principle

The implementation principle of the all-optical OR gate is relatively straightforward. Its core operation relies primarily on the gain saturation and XPM effects in a SOA under optical signal injection. A schematic diagram of the OR gate implementation using a SOA-DI is shown in Figure 4 [37]. Data signals A and B, along with a CW beam, are combined and injected into an SOA, followed by a DI to perform the OR operation. Inside the CR-SOA, signals A and B utilize XPM to induce a phase change in the CW signal. After leaving the SOA, the CW signal is split into two parts by a 3 dB OC and then enters the DI, where the delay time ($\Delta_{\tau }$) and phase bias ($\Delta_{\emptyset }$) are adjusted to create a phase difference between the CW components. When the input signal combination is (A = B = “1”/A = “0”, B = “1”/A = “1”, B = “0”), the CW components will fall within the created phase window and are forwarded at the DI output, resulting in a logic “1”. On the other hand, those CW components that do not experience a phase shift are pushed outside the phase window and suppressed at the DI output, yielding a “0” [99]. The OR truth table is shown in Table 1c.

#### 4.3.2. OR Logic Gate Technological Development-Based SOAs

**Figure 14:**
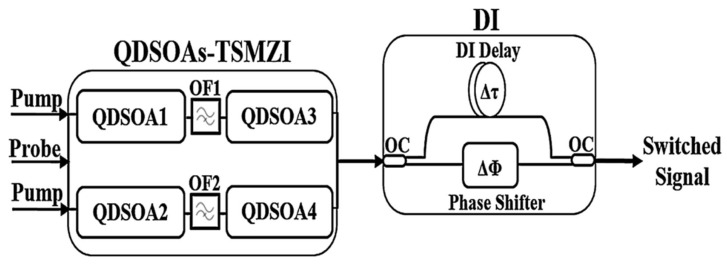
Schematic diagram of QDSOAs-TSMZI with DI. OF: optical filter. OC: 3 dB optical coupler [107], “reprinted with permission from Ref. [107], copyright (2020), Elsevier”.

Conventional bulk and quantum well SOAs are the basic devices for realizing the OR gate in the early stage. Their XGM scheme has a simple structure, but is limited by the carrier recovery time, and the typical working rate is usually within the range of 10–40 Gb/s. Due to the influence of ASE noise, the ER is usually low. One study [100] achieved all-optical OR operation at 20 Gb/s and 40 Gb/s based on SOA-DI, revealing that the operation rate is limited by the carrier lifetime and the input pulse energy. Due to the nonlinear characteristics of optical solutions, the speed can be improved, and one study [101] numerically simulated the SOA-DI-based solutions all-optical OR gate at 80 Gb/s, achieving logical correctness and high QF. One study [102] conducted numerical simulation at the same rate, using a dual-probe SOA-MZI to implement the all-optical OR gate, and the proposed scheme can achieve higher QF and less mode dependence compared to SOA-DI. With the development of TPA technology, researchers successfully simulated the SOA-DI all-optical OR gate based on TPA at 250 Gb/s [103]. QD-SOA, with its unique discrete energy states and ultrafast carrier dynamics, has raised the rate of the SOA-MZI scheme to above 100 Gb/s. Its enhanced gain nonlinearity and lower noise coefficient bring higher ER (10–15 dB) and better signal quality, making it one of the preferred solutions for achieving high-speed and high-performance all-optical OR gates [53]. The TSMZI-QDSOA scheme employs a three-stage cascade collaborative configuration, which integrates dual-stage QD-SOA signal processing modules into both arms of an MZI and further connects a DI in series, thereby significantly enhancing the overall performance of the all-optical logic gate [104,105,106]. The schematic diagram is shown in Figure 14 [107]. Using a QDSOA-assisted TSMZI in serial with a DI, the scheme was demonstrated at 1 Tb/s, achieving better QF. This result proves that TSMZI architecture can increase the operational speed several times that of a conventional single SOA configuration. One study [84] utilized the TPA effect, with a data transmission rate of up to 2 Tb/s, proving that using TPA can achieve QF twice that of the structure of QD-SOA. PhC, due to its slow-light effect that can constrain and control the movement of photons, has been experimentally proven to be able to implement the OR function at high speeds with ultra-low power consumption [108], achieving an extremely high ER, and is highly suitable for high-density, low-power photonic integrated circuits [65]. RSOA, due to its compact unidirectional input–output structure, achieves performance superior to conventional SOA at high speeds [109] and demonstrates high integration friendliness when implementing the OR gate based on XGM, having unique advantages in compact modules and low-cost solutions. By applying the TPA mechanism to the CR-SOA-DI configuration, the operational speed for the OR gate was substantially increased from 100 Gb/s to 320 Gb/s, while the QF experienced a modest reduction from 9 [99] to 7.85 [98], highlighting the effectiveness of the nonlinear enhancement for speed scaling despite a compromise in signal quality.

### 4.4. Implementation of NOR Gate

#### 4.4.1. Operation Principle

The all-optical NOR gate, being a universal logic gate capable of constructing any logic function, typically requires more complex implementation schemes than the OR gate. Therefore, it often necessitates the combined use of XGM and XPM effects and relies more heavily on the interferometer structures to achieve precise phase-to-intensity conversion, thereby attaining a high ER and a robust noise immunity. The operation principle of the NOR gate based on an SOA-MZI is illustrated in Figure 5 [38]. The combined data of signals A and B is injected via a WSC into SOA1 at port 1, while a clock signal is injected into SOA2 at port 2. Simultaneously, a CW beam is split by a 3 dB OC into two equal parts, which enter SOA1 and SOA2 through port 3. Signals A and B modulate the gain and phase of the CW light via the nonlinear effects of XGM and XPM. Consequently, when a high-power signal combination (A = "0", B = “1”/A = “1”, B = “0”/A = “1”, B = “1”) is input into CR-SOA1 and a clock signal (all “1”) is simultaneously input into SOA2, both SOAs reach saturation. As a result, the modulated phases of the probe signals from the two arms after passing through their respective CR-SOAs become identical, leading to destructive interference at the MZI output and thus producing a logic ‘0’. If a low-power signal combination (A = “0”, B = “0”) is input, the injection of the clock signal disrupts the phase balance of the MZI. This results in constructive interference at the MZI output, yielding a logic ‘1’. In this way, the Boolean NOR gate is realized. The corresponding truth table is shown in Table 1d.

#### 4.4.2. NOR Logic Gate Technological Development-Based SOAs

When implementing NOR gates using conventional bulk and quantum well SOAs, the XPM scheme based on SOA-MZI is commonly adopted. This scheme has a relatively mature structure, but is limited by the carrier recovery time, resulting in a rate generally ranging from 10 to 40 Gb/s. It is also sensitive to input power and polarization state, with an ER typically within the range of 8–12 dB. There is also a significant code type effect. In the study [110], a soliton all-optical NOR gate based on SOA-MZI was theoretically analyzed at 80 Gb/s, demonstrating logical correctness and high QF. Employing the TPA effect in SOAs-MZI configuration enabled XOR operation at up to 250 Gb/s, yielding a QF of 9.6 [111]. QD-SOA, with its ultrafast carrier dynamics and enhanced nonlinear refractive index change, shows significant advantages in the implementation of NOR gates based on MZI, capable of supporting working rates far exceeding those of conventional devices. Experimental studies have shown that using QD-SOA-MZI can achieve NOR logic operations at up to 160 Gb/s or even higher rates, while obtaining excellent ER (>12 dB) and low BER. This is mainly due to the picosecond-level recovery time effectively suppressing inter-symbol interference [112]. Meanwhile, the schemes based on TS and TPA can outperform conventional SOA schemes in various indicators, with rates even reaching the Tb/s level [107,113]. The PhC-SOA implementation of NOR gates mainly utilizes its extremely strong slow-light-enhanced nonlinear effect, allowing for the generation of sufficient nonlinear phase shifts at very low-control light powers, enabling NOR functionality to be realized in photonic crystal waveguide interferometers with ultra-low power consumption. It has an irreplaceable potential in the design of logic units for high-density, low-power photonic integrated circuits. One study [114] numerically simulated 160 Gb/s all-optical NOR and XNOR logic gates based on PhC-SOA-MZI, by examining the changes in QF with key operating parameters, including the influence of ASE and working temperature. It was found that the performance was superior to that of conventional SOA. Due to RSOA’s high gain and low noise at low driving currents, the study [109] numerically analyzed the ultrafast performance of all-optical NOR and XNOR logic gates at 120 Gb/s using a dual RSOA scheme, and experimentally proved that RSOA can achieve more acceptable performance at the target rate and make the implementation of the logic gate more feasible. The TPA-enhanced CR-SOA-MZI scheme achieved a significantly higher speed of 320 Gb/s for the NOR operation, compared to 120 Gb/s of its standard counterpart, with a corresponding decrease in QF from 14 [98] to 12.12 [115], illustrating the characteristic speed-signal integrity trade-off at ultra-high bitrates.

### 4.5. Implementation of XOR Gate

#### 4.5.1. Operation Principle

The all-optical exclusive-OR (XOR) serves as the core logic unit for implementing arithmetic operations, data comparison, and encryption/decryption. The logical requirement of the XOR gate is output is “1” when the inputs differ. This usually requires precise interference cancellation or nonlinear signal cancellation mechanisms to achieve, so the mainstream solutions highly rely on interferometer structures based on XPM/FWM effects. These solutions impose strict requirements on the nonlinear phase shift efficiency, recovery speed, and stability of the SOA. The schematic diagram illustrating the principle of the XOR operation is shown in Figure 6 [18]. Data signals A ($\lambda_{1}$) and B ($\lambda_{2}$) are injected into the two arms of the MZI through ports 1 and 2, respectively. Simultaneously, 3 dB OC splits a CW ($\lambda_{CW}$) beam, which acts as the probe, into two parts, directing them into SOA1 and SOA2. The combination of signals A and B then induces phase changes in the CW signal within their respective SOA branches. Specifically, when A = “0” and B = “0”, the MZI remains balanced, yielding no output and a logical result of “0”. When A = “1” and B = “0”, the CW signal branch propagating in the upper arm of the MZI, along with signal A, compared with the other split path signals propagating in the lower arm of the MZI, will undergo phase changes through XPM. Therefore, when these CW branches are recombined, they will undergo constructive interference, producing a “1” output. The same situation occurs when A = “0” and B = “1”. However, when A = “1” and B = “1”, the phase changes introduced by these CW branches in the two MZI arms cancel each other, resulting in destructive interference and a final output of “0”, thereby achieving the XOR logic operation. The result is imprinted on the output light at a specific wavelength ($\lambda_{CW}$), which is selected by an OBPF [116]. The corresponding truth table is shown in Table 1e.

#### 4.5.2. XOR Logic Gate Technological Development-Based SOAs

When implementing the XOR gate using conventional bulk and quantum well SOA, the SOA-MZI structure is often adopted. However, due to the carrier recovery time limitation, the rate is usually within the range of 10–40 Gb/s, and it is sensitive to input power and polarization state fluctuations, with significant code type effects, which limit its application in high-speed systems [117,118,119,120]. With the advancement of technology, the studies in [121,122] demonstrated the implementation of 20 GHz full duty cycle operation and 20 Gb/s pseudo-data mode-based all-optical Boolean XOR logic using SOA-assisted Sagnac switch, accompanied by low signal energy and negligible code type effects. High-quality all-optical XOR operation at 86.4 Gb/s was demonstrated using a hybrid photonic integrated device, effectively mitigating the patterning effect caused by the slow carrier recovery of SOAs [123]. Experiment [124] combined MZI and DI to improve pulse quality. One study [125,126,127,128] utilized a single SOA’s UNI to achieve the XOR function, which is of practical significance for simplifying and assisting the design of more complex interconnection systems with XOR gates as the core logic unit. Meanwhile, in the SOA-integrated MZI structure, based on the TPA effect [129,130] and soliton characteristics [131], high-speed XOR logic gates were also achieved. QD-SOA, with its ultrafast carrier dynamics and enhanced nonlinear coefficient, becomes the preferred choice for realizing ultra-high-speed XOR gates. The MZI scheme based on QD-SOA has successfully demonstrated XOR operations from 160 Gb/s to over 1 Tb/s, with picosecond-scale phase shift recovery time effectively suppressing inter-symbol interference, while achieving a high ER and good signal integrity, providing key device support for the next-generation ultra-high-speed all-optical signal processing [132,133,134,135]. Additionally, based on the realization of ultra-high-speed XOR logic gates using the TPA effect [136], researchers integrated QDSOA and TS-MZI structures to further enhance speed and performance. PhC-SOA’s XOR gate is mainly attributed to its extremely strong slow-light enhancement nonlinear effect, enabling the generation of the required π phase shift at extremely low-control light power, thus achieving XOR logic in an interferometer-based optical waveguide with ultra-low power consumption, outperforming conventional SOA [59]. Although its working rate is limited by the slow-light bandwidth, it can achieve extremely high ER and excellent energy efficiency ratio, making it a highly promising solution for logic units in high-density photonic integrated circuits. The compact cavity of RSOA enhances nonlinear effects for XPM-based XOR gates, offering integrated and low-cost solutions. A notable demonstration using dual RSOAs achieved 120 Gb/s operation with a high QF of 35, highlighting its exceptional signal integrity [137]. While the standard CR-SOA-MZI XOR gate achieved a QF of 18.5 at 100 Gb/s [116], its TPA-enhanced counterpart reached a significantly higher speed of 320 Gb/s at the expense of a notable reduction in signal quality, with the QF decreasing to 9.26 [98].

### 4.6. Implementation of XNOR Gate

#### 4.6.1. Operation Principle

The all-optical XNOR gate, as the logical complement of the XOR gate, serves as another key unit for implementing arithmetic operations, data comparison, and pattern recognition. This characteristic leads to implementation schemes that are often highly symmetric with the XOR gate, primarily based on XPM structures with an interferometer. The XNOR function can be realized either by utilizing the complementary output port of the interferometer or by simply applying optical inversion to the XOR output, which again demonstrates the flexibility of interferometric schemes in achieving reconfigurable logic functions. As shown in Figure 7 [38], where one data input is replaced by a Clk signal, the INVERT operation is analogous to the XOR operation. When the output from the XOR gate and the Clk signal are fed into SOA3 and SOA4, respectively, to perform the INVERT operation, a CW beam is simultaneously injected into the central section of the SOAs-MZI2. Consequently, the result of the A XNOR B logic operation is obtained from the OC at port 8 [87]. Its corresponding truth table is presented in Table 1f.

#### 4.6.2. XNOR Logic Gate Technological Development-Based SOAs

The conventional bulk and quantum well SOA for implementing the XNOR gate usually adopts the MZI structure. The XNOR function is obtained by directly monitoring the other output port of the interferometer. Although this scheme is straightforward, it is limited by the carrier recovery time, with the rate typically being 10–40 Gb/s. Moreover, due to the limited interference contrast and the influence of noise, the ER is generally within the range of 8–12 dB, requiring high stability of the operating point. One study [138] numerically simulated the performance of SOA-MZI at 80 Gb/s, focusing on analyzing the influence of ASE and input pulse energy on the QF, and found that the high spontaneous emission factor and high pulse energy led to QF reduction. One study [139] studied the performance of the all-optical XNOR gate based on TPA at a rate of 250 Gb/s, proving its logical correctness and sufficient QF under the consideration of ASE influence. QD-SOA, with its ultrafast carrier dynamics and high nonlinear refractive index change, can simultaneously provide high-quality XOR and XNOR complementary outputs in the MZI-based scheme. Experiments have confirmed that using a single QD-SOA can achieve XNOR operations at rates up to 160 Gb/s or even higher, with its excellent phase shift recovery characteristics ensuring good ER and low BER at high bit rates, making it a core technology for building ultra-high speed all-optical arithmetic logic units [140]. Based on the QD-SOA-MZI structure, combined with the TPA effect, even Tb/s rates have been achieved, and the QF can be significantly improved [141]. PhC-SOA enables the implementation of an XNOR gate primarily by leveraging the pronounced nonlinear enhancement from the slow-light effect. This allows for the generation of a precise π phase shift within the interferometer-based optical waveguide at an extremely low control power, thereby facilitating ultra-low-power-consumption XNOR logic. Leveraging this mechanism in a PhC-SOA-MZI configuration under XPM, an operational speed of 160 Gb/s was achieved while maintaining a QF of 15.83 [114]. CR-SOA, through its optimized carrier recovery process, can provide more consistent phase shifts and purer output signals than conventional SOA at medium to high rates when used in interferometer-type XNOR gates. The TPA-enhanced CR-SOA-MZI scheme notably increased the operational speed from 120 Gb/s to 320 Gb/s. At the same time, the QF experienced a slight reduction from 12.4 [87] to 10.78 [98], demonstrating a trade-off between speed and signal integrity in ultra-high-bitrate operations.

## 5. Comparison and Integrated Progress

### 5.1. Comparison of Key Performance Indicators

Table 2, Table 3, Table 4, Table 5, and Table 6 summarize the key performance indicators (such as operating speed and QF) reported for all-optical logic gates (XOR, AND, OR, NOR, NAND, and XNOR) implemented using five SOA-based device architectures (SOA, QD-SOA, PhC-SOA, RSOA, and CR-SOA) under different schemes, with a clear distinction between experimental (Exp.) and numerical simulation (Sim.) results.

**Table 2:** Key performance indicators based on SOA schemes.

Logic Gate	Scheme	Speed	QF	Result Type (Exp./Sim.)	References
XOR	SOAs-MZI	10–100 Gb/s	-	Exp., Exp., Exp., Exp., Exp.	[4,118,119,120,124]
	SOA-UNI	20 Gb/s	-	Sim.	[127]
	SOA-UNI-DI	40 Gb/s	6	Sim., Sim.	[125,126]
	PSK (SOAs-MZI)	100 Gb/s	20	Sim.	[117]
	Soliton (SOAs-MZI)	80 Gb/s	24.83	Sim.	[131]
	PC-SOAs-MZI	160 Gb/s	21	Sim.	[59]
	TPA (SOAs-MZI)	250 Gb/s	11	Exp., Sim.	[129,130]
AND	XPM (SOAs-MZI)	80 Gb/s	16.65	Sim., Exp. and Sim., Exp.	[76,77,78]
	TPA (SOAs-MZI)	250 Gb/s	10.8	Exp., Exp.	[92,93]
	Soliton (SOAs-MZI)	80 Gb/s	14.41	Sim.	[79]
OR	SOA-DI	20–80 Gb/s	8.5–12.05	Sim., Exp. and Sim., Exp.	[76,77,100]
	Soliton (SOA-DI)	80 Gb/s	21.22	Sim.	[102]
	TPA (SOA-DI)	250 Gb/s	-	Sim.	[103]
NOR	XGM (a SOA)	10 Gb/s	-	Exp.	[88]
	SOA-OBF	40 Gb/s	-	Exp.	[36]
	XPM (SOAs-MZI)	80 Gb/s	12.05	Exp., Sim.	[4,38]
	Soliton (SOAs-MZI)	80 Gb/s	24.85	Sim.	[110]
	TPA (SOAs-MZI)	250 Gb/s	9.6	Sim.	[111]
NAND	XGM(a SOA)	10 Gb/s	-	Exp.	[88]
	XPM (SOAs-MZI)	80 Gb/s	-	Exp.	[4]
	SOA-DI	80 Gb/s	10.75	Exp.	[90]
	TPA (SOAs-MZI)	250 Gb/s	6.7	Exp., Exp.	[92,93]
XNOR	SOA-OBF	40 Gb/s	-	Exp.	[36]
	XPM (SOAs-MZI)	80 Gb/s	10.13	Sim., Exp., Sim.	[76,123,138]
	TPA (SOAs-MZI)	250 Gb/s	12.34	Sim.	[139]

**Table 3:** Key performance indicators based on QD-SOA schemes.

Logic Gate	Scheme	Speed	QF	Result Type (Exp./Sim.)	References
XOR	QD-SOAs-MZI	250 Gb/s	-	Sim., Sim., Sim.	[82,132,135]
	QD-SOAs-OBF	160 Gb/s	21	Sim.	[134]
	TPA (QD-SOAs-MZI)	2 Tb/s	14	Sim.	[136]
	XPM (QD-SOAs-TS-MZI)	1 Tb/s	18.5	Sim.	[83]
AND	XGM/XPM (QD-SOAs-MZI)	250 Gb/s	14	Sim., Sim.	[82,132]
	TPA (QD-SOAs-MZI)	2 Tb/s	17	Sim.	[84]
	XPM (QD-SOAs-TS-MZI)	1 Tb/s	13.6	Sim.	[83]
OR	XGM/XPM (QD-SOA-DI)	250 Gb/s	9	Sim., Exp.	[82,132]
	TPA (QD-SOA-DI)	2 Tb/s	15	Sim.	[84]
	XPM (QD-SOAs-TSMZI-DI)	1 Tb/s	14	Sim.	[107]
NOR	XPM (QD-SOAs-MZI)	1 Tb/s	40	Sim.	[112]
	TPA (QD-SOAs-MZI)	2 Tb/s	9.6	Sim.	[113]
	XPM (QD-SOAs-TS-MZI)	1 Tb/s	14	Sim.	[107]
NAND	XPM (QD-SOAs-MZI)	160 Gb/s	40	Sim.	[94]
	TPA (QD-SOAs-MZI)	2 Tb/s	13	Sim.	[84]
XNOR	XPM (QD-SOAs-MZI)	160 Gb/s	29.72	Sim.	[140]
	TPA (QD-SOAs-MZI)	1 Tb/s	31	Sim.	[141]
	TPA (QD-SOAs-MZI)	2 Tb/s	9.8	Sim.	[113]

**Table 4:** Key performance indicators based on PhC-SOA schemes.

Logic Gate	Scheme	Speed	QF	Result Type (Exp./Sim.)	References
XOR	XPM (PhC-SOAs-MZI)	160 Gb/s	20	Sim.	[59]
AND	XPM (PhC-SOAs-MZI)	160 Gb/s	35.87	Sim.	[108]
OR	XPM (PhC-SOA-DI)	160 Gb/s	23	Sim.	[108]
NOR	XPM (PhC-SOAs-MZI)	160 Gb/s	20.1	Sim.	[114]
NAND	XPM (PhC-SOAs-MZI)	160 Gb/s	18	Sim.	[96]
XNOR	XPM (PhC-SOAs-MZI)	160 Gb/s	15.83	Sim.	[114]

**Table 5:** Key performance indicators based on RSOA schemes.

Logic Gate	Scheme	Speed	QF	Result Type (Exp./Sim.)	References
XOR	XPM (dual-RSOA)	120 Gb/s	35	Sim.	[137]
AND	RSOAs-MZI	120 Gb/s	-	Sim.	[86]
OR	RSOA-DI	120 Gb/s	-	Sim.	[86]
NOR	RSOAs-MZI	120 Gb/s	-	Sim.	[109]
NAND	RSOAs-MZI	120 Gb/s	-	Sim.	[97]
XNOR	XPM (RSOAs-MZI)	160 Gb/s	15.83	Sim., Sim.	[97,109]

**Table 6:** Key performance indicators based on CR-SOA schemes.

Logic Gate	Scheme	Speed	QF	Result Type (Exp./Sim.)	References
XOR	CR-SOAs-MZI	100 Gb/s	18.5	Sim.	[116]
	TPA (CR-SOAs-MZI)	320 Gb/s	9.26	Sim.	[98]
AND	CR-SOAs-MZI	100 Gb/s	14	Sim.	[73]
	TPA (CR-SOAs-MZI)	320 Gb/s	14.82	Sim.	[98]
OR	CR-SOAs-DI	100 Gb/s	9	Sim.	[99]
	TPA (CR-SOAs-DI)	320 Gb/s	7.85	Sim.	[98]
NOR	CR-SOAs-MZI	120 Gb/s	14	Sim.	[115]
	TPA (CR-SOAs-MZI)	320 Gb/s	12.12	Sim.	[98]
NAND	CR-SOAs-MZI	120 Gb/s	13.5	Sim.	[87]
	TPA (CR-SOAs-MZI)	320 Gb/s	15.64	Sim.	[98]
XNOR	CR-SOAs-MZI	120 Gb/s	12.4	Sim.	[87]
	TPA (CR-SOAs-MZI)	320 Gb/s	10.78	Sim.	[98]

### 5.2. Integrated Attempts for Photonic Integrated Circuits

The key step for the all-optical logic gates to move from the experimental platform of discrete optical fibers towards practical application lies in their integration with PICs technology. Integration not only significantly reduces the size, power consumption and cost of the system, but also enhances stability and reliability, which is the inevitable path to achieving complex all-optical signal processing functions. For all-optical logic-based SOAs, the integration attempts mainly follow three paths: compact and multifunctional integration for specific devices, such as integration-based RSOA/PhC-SOA; monolithic integration on the native active material platform of InP; through hybrid or heterogeneous integration technologies on the dominant silicon-based photonic platform, introducing the nonlinear function of SOA.

#### 5.2.1. Compact and Multifunctional Integration

With its high integration-friendliness brought by the reflective cavity structure, RSOA has become one of the key active devices for achieving compact and multifunctional photonic chips. The characteristic that the optical signal is input and output from the same end face greatly simplifies the coupling design with planar optical wave circuits, avoiding the complex multiport alignment issues. This enables RSOA to be efficiently embedded in structures such as MZI, ring resonators, or multimode interference couplers on the chip, serving as the core unit for tunable gain and nonlinear phase shift. Studies have shown that by using this integrated RSOA unit, functional modules for high-speed all-optical signal processing can be successfully constructed [142]. Such solutions open feasible technical pathways for achieving high-density, low-cost photonic information processing chips, especially in scenarios requiring reconfigurable logic and wavelength flexibility in access networks and data center optical interconnections. PhC-SOA represents the direction of achieving disruptive integration through structural innovation. It utilizes the slow-light effect of photonic crystals to generate strong nonlinear interactions within the micrometer-scale device length, providing the possibility for achieving ultra-high density and ultra-low power consumption all-optical logic chips. Although its preparation process is complex and coupling challenges are significant, it has been proven to be capable of single-chip integration with other photonic crystal components to achieve complete signal processing functions [143].

#### 5.2.2. Monolithic Integration Based on InP Material

In terms of material platform-level monolithic integration, the QD-SOA monolithic integration based on the InP material is the most direct approach. InP, as the representative of the III-V group compound semiconductors, is an ideal substrate for the fabrication of active optical devices such as lasers, amplifiers, and high-speed modulators. Its bandgap perfectly matches the optical communication band, and it has excellent electronic transport properties. On this platform, QD-SOA can be monolithically integrated with passive waveguides, power dividers/couplers, phase modulators, and even photodetectors on the same chip, thereby constructing a complete and compact all-optical signal processing subsystem. This monolithic integration scheme can maximize the unique physical advantages of quantum dot materials. The three-dimensional quantum confinement effect resulting in ultrafast carrier dynamics (ps recovery time) and a wide gain spectrum enables the QD-SOA integrated on the chip to achieve core processing functions such as high-speed logic operations, wavelength conversion, and signal regeneration at hundreds of gigabits per second, providing a material basis for the development of dedicated all-optical coprocessor chips [144]. However, this technical path still faces core challenges for large-scale practical application. The primary challenge lies in material growth, that is, how to epitaxially grow a highly uniform, high-density, and optically high-quality quantum dot gain region on the InP substrate, which directly determines the consistency and reproducibility of the device performance. Secondly, integrating multiple functionally distinct active and passive devices monolithically has extremely high process complexity, involving multiple epitaxial growths, fine etching, and electrical isolation steps, resulting in increased manufacturing costs and strict requirements for process tolerances. Nevertheless, research progress has preliminarily verified its feasibility. By integrating multiple sections of SOA with different functions on the same InP chip and combining array waveguide grating and other passive components, basic processing functions, including multiple logic gates, have been successfully demonstrated [145]. These works have laid important process and design foundations for the future realization of a complete on-chip optical processor integrating complex logic units, reconfigurable optical switch arrays, and cascaded regenerators, and pointed out the direction of overcoming existing bottlenecks through continuous innovation in materials and integration processes.

#### 5.2.3. Silicon-Based Hybrid Integration and Heterogeneous Integration

Silicon-based photonics, due to its high compatibility with complementary metal oxide semiconductor (CMOS) technology, extremely high integration density, and significant cost advantages brought by large-scale manufacturing, has become the dominant platform for developing PICs. However, silicon itself is an indirect bandgap material with low light emission efficiency, making it difficult to directly fabricate high-performance SOAs. Therefore, the combination of SOA-based all-optical logic with the silicon photonic platform mainly relies on hybrid integration or hetero-integration technologies. Hybrid integration involves bonding the SOA (such as bulk SOA/QD-SOA) prepared on III-V-based chips (e.g., InP) through precise flip-chip bonding or micro-transfer printing techniques onto the silicon chip that has already been fabricated with silicon photonic waveguides, couplers, and interferometers. This integration method combines the excellent gain characteristics of III-V materials with the high integration advantages of the silicon photonic platform, and is currently a relatively mature solution, having been used for demonstrating integrated wavelength conversion and logic gates [146]. Hetero-integration occurs at the wafer level, through epitaxial growth or direct bonding, integrating the III-V gain materials with the silicon waveguide core layer on the same substrate, and then directly fabricating SOA devices based on silicon waveguides through processes such as lithography [147]. This method enables more compact integration and lower coupling losses and is a long-term development direction for large-scale production. Despite challenges such as material lattice mismatch and differences in thermal expansion coefficients, significant progress has been made in this field, laying the foundation for the realization of truly large-scale, low-cost silicon-based all-optical computing chip technology in the future.

## 6. Challenges and Future Prospects

Although SOA-based all-optical logic gate technologies have achieved remarkable progress in operating speed, functional diversity, and integration capability, several critical challenges remain before large-scale and practical deployment can be realized [148]. To provide clearer prioritization and actionable insight, the challenges and prospects are discussed below from device-level physical, integration-level, and system-level perspectives, with emphasis on architecture-dependent issues.

### 6.1. Device-Level Physical Challenges

At the device level, the dominant challenges are associated with ultrafast carrier dynamics, noise accumulation, thermal effects, and polarization sensitivity [149]. While QD-SOAs and CR-SOAs exhibit significantly shortened carrier recovery times compared with conventional bulk SOAs, further optimization is still required to fully suppress pattern-dependent effects and achieve distortion-free operation at ultra-high bit rates. In particular, ASE noise and carrier-density fluctuations remain major limiting factors for cascaded logic operations. Thermal effects under high optical-pumping conditions introduce gain drift and phase instability, especially in interferometric configurations such as SOA-MZIs and RSOA-MZIs. In addition, polarization sensitivity in nonlinear processes continues to restrict system robustness, particularly for PhC-SOAs and conventional SOAs operating without polarization management.

### 6.2. Integration and Fabrication Challenges

At the integration level, process complexity, fabrication tolerance, and crosstalk management represent key obstacles [150]. As multiple logic units are integrated onto a single PIC, inter-device crosstalk and thermal coupling become increasingly severe, requiring advanced waveguide engineering and isolation strategies. Heterogeneous integration across different material platforms—such as III–V/Si photonics—faces challenges related to coupling loss, alignment tolerance, and yield variability, which directly impact system stability and scalability [151]. PhC-SOAs, in particular, demand nanometer-scale fabrication precision, while QD-SOAs require strict control of quantum dot size uniformity and epitaxial quality. Furthermore, on-chip testing and packaging technologies must evolve to support large-scale, high-density SOA-based PICs.

### 6.3. System-Level and Network-Level Challenges

From a system perspective, power consumption, scalability, and cascading performance are the most critical limitations [152]. Despite continuous improvements, the energy consumption of current all-optical logic solutions remains significantly higher than that of electronic logic gates, limiting their applicability in large-scale computing systems. Performance degradation due to noise accumulation and pattern effects restricts the depth of cascaded logic operations, particularly in conventional SOAs and RSOAs. Moreover, compatibility with existing optical communication infrastructures—including wavelength grids, modulation formats, and network protocols—must be ensured to enable seamless integration into future optical networks.

### 6.4. Architecture-Specific Perspectives and Research Roadmap

From an architecture-specific standpoint, QD-SOAs and CR-SOAs currently offer the most promising balance between speed, noise tolerance, and cascading capability due to their favorable carrier dynamics and reduced pattern effects [72,73]. These architectures are well-suited for near-term deployment in high-speed (>100 Gb/s) all-optical logic and signal processing systems. PhC-SOAs provide a compelling pathway toward ultra-low-power and ultra-compact logic devices by exploiting slow-light-enhanced nonlinear interactions [61,62,63,64,65,66,67,68]. However, mid-term research efforts must prioritize bandwidth management, fabrication tolerance, and dispersion control to fully unlock their potential. Conventional SOAs and RSOAs remain attractive for cost-effective and integration-friendly implementations but require continued advances in noise suppression, thermal management, and system-level optimization to support complex logic circuits. In the longer term, the convergence of SOA-based logic devices with emerging nanophotonic and programmable photonic platforms, including nonlinear photonic-crystal nanocavities, metasurface-assisted structures, and large-scale reconfigurable photonic integrated circuits, offers new opportunities to enhance functional density, energy efficiency, and system scalability [153,154,155]. Building on these advances, the integration of SOA-based logic devices with emerging computing paradigms, particularly neuromorphic photonics, represents a promising research direction. By exploiting the intrinsic nonlinear dynamics of SOAs to emulate neuronal excitation and inhibition behaviors, all-optical neuromorphic hardware may extend beyond the limitations of traditional Boolean logic and enable new functionalities in optical computing, intelligent communication systems, and high-speed optical sensing networks [156,157].

## 7. Conclusions

This review systematically explores the technological progress of all-optical logic gates (AND, NAND, OR, NOR, XOR, and XNOR) based on SOA and its derivative structures. The research shows that the technological evolution from conventional bulk and quantum well SOA to QD-SOA, PhC-SOA, RSOA, and CR-SOA essentially involves regulating carrier dynamics and optical field confinement through material engineering and structural innovation, thereby overcoming the bottlenecks of slow recovery time and insufficient nonlinear efficiency in conventional SOAs. These devices, with their unique physical mechanisms and structural advantages, such as the ultrafast carrier relaxation of QD-SOAs, the slow-light enhancement nonlinearity of PhC-SOA, and the integration friendliness of RSOA, provide diverse physical solutions for the realization of basic Boolean logic functions. They have successfully increased the operation rate from 10 Gb/s to the Tb/s scale in experiments and continuously optimized key indicators such as ER and QF. Comprehensive analysis indicates that there is no universal “best” device, and a trade-off selection needs to be made based on the target logic function, rate, power consumption, and integration platform. For example, the ultra-high-speed XOR gate often adopts the interferometer scheme based on QD-SOA, while low-power compact integration may prefer the XGM scheme based on RSOA. Although there are still continuous challenges in fundamental physics, integration, and system-level challenges, in the future, through deep integration of new materials, new mechanisms, and combinations with emerging directions such as neuromorphic computing, all-optical logic technology is expected to move from discrete gate-level demonstrations to complex, reconfigurable, and system-level photonic integrated circuits, ultimately providing practical hardware solutions for breaking through the electronic bottleneck in all-optical information processing and laying the foundation for future all-optical networks and optical computing.

## Figures and Tables

**Table 1:** Truth table of all-optical logic gates (**a**) AND, (**b**) NAND, (**c**) OR, (**d**) NOR, (**e**) XOR, and (**f**) XNOR.

Input A	Input B	Output Y	Input A	Input B	Output Y
(**a**) AND gate			(**b**) NAND gate		
0	0	0	0	0	1
0	1	0	0	1	1
1	0	0	1	0	1
1	1	1	1	1	0
(**c**) OR gate			(**d**) NOR gate		
0	0	0	0	0	1
0	1	1	0	1	0
1	0	1	1	0	0
1	1	1	1	1	0
(**e**) XOR gate			(**f**) XNOR gate		
0	0	0	0	0	1
0	1	1	0	1	0
1	0	1	1	0	0
1	1	0	1	1	1

## Data Availability

Data are contained within the article.

## Abbreviations

SOA	Semiconductor Optical Amplifier
QD-SOA	Quantum Dot Semiconductor Optical Amplifier
PhC-SOA	Photonic Crystal Semiconductor Optical Amplifier
RSOA	Photonic Crystal Semiconductor Optical Amplifier
CR-SOA	Carrier Reservoir Semiconductor Optical Amplifier
MZI	Mach–Zehnder Interferometer
TS-MZI	Turbo-Switched Mach–Zehnder Interferometer
XOR	Exclusive-OR
QF	Quality Factor
BER	Bit Error Rate
ER	Extinction Ratio
XGM	Cross-Gain Modulation
XPM	Cross-Phase Modulation
FWM	Four-Wave Mixing
TPA	Two-Photon Absorption
ASE	Amplified Spontaneous Emission
CR	Carrier Reservoir
AR	Active Region
CW	Continues Wave
OBPF	Optical Bandpass Filter
OC	Optical Coupler
WSC	Wavelength-Selective Coupler
RZ	Return-to-Zero
DI	Delayed Interferometer
